# Microfluidic-based human prostate-cancer-on-chip

**DOI:** 10.3389/fbioe.2024.1302223

**Published:** 2024-01-23

**Authors:** Linan Jiang, Hunain Khawaja, Shekha Tahsin, Tanjia A. Clarkson, Cindy K. Miranti, Yitshak Zohar

**Affiliations:** ^1^ Department of Aerospace and Mechanical Engineering, Tucson, AZ, United States; ^2^ Cancer Biology Graduate Interdisciplinary Program, Tucson, AZ, United States; ^3^ College of Sciences, Tucson, AZ, United States; ^4^ Department of Molecular and Cellular Biology, Tucson, AZ, United States; ^5^ University of Arizona Cancer Center, University of Arizona, Tucson, AZ, United States

**Keywords:** organ-on-chip, microfluidics, prostate cancer, stromal fibroblasts, cancer-associated fibroblasts (CAFs), prostate tumor invasion, tumor microenvironment

## Abstract

Lack of adequate models significantly hinders advances in prostate cancer treatment, where resistance to androgen-deprivation therapies and bone metastasis remain as major challenges. Current *in vitro* models fail to faithfully mimic the complex prostate physiology. *In vivo* animal models can shed light on the oncogenes involved in prostate cancer development and progression; however, the animal prostate gland is fundamentally different from that of human, and the underlying genetic mechanisms are different. To address this problem, we developed the first *in vitro* microfluidic human Prostate-Cancer-on-Chip (PCoC) model, where human prostate cancer and stromal fibroblast cells were co-cultivated in two channels separated by a porous membrane under culture medium flow. The established microenvironment enables soluble signaling factors secreted by each culture to locally diffuse through the membrane pores affecting the neighboring culture. We particularly explored the conversion of the stromal fibroblasts into cancer-associated fibroblasts (CAFs) due to the interaction between the 2 cell types. Immunofluorescence microscopy revealed that tumor cells induced CAF biomarkers, αSMA and COL1A1, in stromal fibroblasts. The stromal CAF conversion level was observed to increase along the flow direction in response to diffusion agents, consistent with simulations of solute concentration gradients. The tumor cells also downregulated androgen receptor (AR) expression in stromal fibroblasts, while an adequate level of stromal AR expression is maintained in normal prostate homeostasis. We further investigated tumor invasion into the stroma, an early step in the metastatic cascade, in devices featuring a serpentine channel with orthogonal channel segments overlaying a straight channel and separated by an 8 µm-pore membrane. Both tumor cells and stromal CAFs were observed to cross over into their neighboring channel, and the stroma’s role seemed to be proactive in promoting cell invasion. As control, normal epithelial cells neither induced CAF conversion nor promoted cell invasion. In summary, the developed PCoC model allows spatiotemporal analysis of the tumor-stroma dynamic interactions, due to bi-directional signaling and physical contact, recapitulating tissue-level multicellular responses associated with prostate cancer *in vivo*. Hence, it can serve as an *in vitro* model to dissect mechanisms in human prostate cancer development and seek advanced therapeutic strategies.

## 1 Introduction

Prostate cancer is the most commonly diagnosed type of cancer (excluding skin cancer) and the second leading cause of cancer death in men in the United State. The standard of care for advanced bone metastatic prostate cancer is androgen deprivation therapy (ADT); however, therapeutic resistance inevitably develops over 18 months (Nakazawa et al., 2017; Kim et al., 2013; Toivanen and Shen, 2017). While identifying and developing new medical targets and therapies is an urgent need, a major barrier to such efforts is primarily attributed to the lack of human relevant prostate cancer models. As the interaction between the epithelium and surrounding stroma is necessary for maintaining normal prostate functions, experimental results confirmed that abnormalities in both the epithelial and the stromal microenvironments are required for prostatic carcinogenesis (Thompson et al., 1989; Lopaczynski et al., 2001). The traditional concept considering stroma as a passive structural element, an idle bystander, has been revised. Stroma in the tumor microenvironment actively participates and contributes to tumorigeneses and cancer progression (Chung et al., 2003; Chung et al., 2005; Pederzoli et al., 2023). Unlike the supportive and protective role of stroma in normal prostate microenvironment, stroma in tumor microenvironment is transformed into a more ‘activated’ phenotype, modifying the stromal extracellular matrix (ECM) and becoming highly proliferative (Pickup et al., 2014). Stromagenesis occurs concurrently with tumorigenesis promoting tumor progression (Beacham and Cukierman, 2005; Castelló-Cros et al., 2009). Communications between the tumor and surrounding stroma are bi-directional through biochemical as well as mechanical signaling. Stroma provides favorable factors facilitating cancer cell growth and survival while cancer cells return the favor by further reactivating the stroma. Moreover, tumor cells invading the surrounding stroma further promote tumor progression mediated by the secretion of favorable factors from both cell types (Friedl and Alexander, 2011; Labernadie et al., 2017; Glentis et al., 2017; Almagro et al., 2022).

Recognizing the important role of stroma in prostate cancer, it is rather a formidable challenge to investigate the tumor-stroma dynamic interactions using traditional cell culture methods. Most *in vitro* prostate cancer models are based on 2D or 3D static cultures of tumor cells, where recapitulation of multicellular interactions between different cell types observed in human tissues is missing. Co-cultures of different cell types in traditional dish cultures often terminate in a few days due to culture medium incompatibility, unsuitable microenvironment for all cell types in the co-cultures, depletion of nutrients, and accumulation of inhibitory biproducts. Attempts to directly mix tumor cells with stroma cells, within the same culture in a dish, inevitably resulted in compromising the individual tissue-level integrity due to missing cell-cell contacts and interactions within the same tissue (Hayward et al., 1995; Olumi et al., 1999; Lopaczynski et al., 2001; Wang et al., 2012; Wang et al., , 2020). To alleviate this problem, Transwells were used to establish two separate tumor and stroma cultures on opposite surfaces of a porous membrane, yet the construction of Transwells is not amenable to microscopic imaging or *in situ* monitoring during experiments. 3D prostate organoids have also been proposed whereby human prostate cancer cell lines, LNCaP and C4-2, were co-cultured independently with prostate or bone stromal cells in a rotating-wall vessel, resulting in increased growth rate, enhanced tumorigenicity and metastatic potential (Jeong et al., 2016; Rhee et al., 2001; Wang et al., 2005). Recent developments in organoid model systems indeed provide additional alternatives for potential high-throughput drug screening tests (Yuki et al., 2020; Hemelryk and Weerden, 2020). Organoids seems to be promising models because they encompass tissue-level multicellular complexity that is physiologically relevant. However, this large degree of complexity poses formidable challenges in probing and assessing the role of individual cell types and the interplays among different types of cells to further dissect molecular mechanisms. Other difficulties encountered in experiments with organoids include maturation, nutrition replenishment, waste removal, and hypoxia (Kim et al., 2020; Andrews and Kriegstein, 2022). Given the inherent tumor-stroma dynamic interplay within the tumor microenvironment, it is highly desirable to dissect the involved interactions revealing the intricate spatiotemporal responses of both tumor and stroma (Sasaki et al., 2017).

Animal models, on the other hand, do not faithfully represent human cancer in many aspects, predominantly due to distinct species differences between animals and humans. More than 90% of cancer drugs, found efficacious in animals, fail in human clinical trials because they are unsafe or ineffective in humans (Seyhan, 2019; Stensland et al., 2021; Stensland et al., 2022; Kim et al., 2022). Consequently, new guidelines of the Food and Drug Administration (FDA) have authorized the use of certain alternatives to animal testing.

Among the alternatives, Organ-on-Chip technology is increasingly recognized for its potential to establishing clinically relevant *in vitro* models (Fong et al., 2016; Vunjak-Novakovic et al., 2021; Baptista et al., 2022; Francis et al., 2022). Organs-on-chips have shown promising functionality in developing disease models for cancer research, e.g., liver chips correctly identified toxicity in 87% of various drugs that passed animal tests, without false identification of nontoxic drugs (Liu et al., 2021; Duzagac et al., 2021; Ingber, 2022; Ewart et al., 2022). Following the Organ-on-Chip concept, we successfully established the first human normal Prostate-on-a-Chip (PoC) model demonstrating the importance of the prostate stroma in inducing and maintaining normal prostate epithelial differentiation. Co-cultured in the Prostate-on-a-Chip, normal stromal fibroblasts secreted androgen-induced morphogens that diffused across a porous separation membrane into the normal epithelium culture resulting in basal to secretory luminal cell differentiation (Jiang et al., 2019). Todate, human *in vitro* models designed to adequately elucidate the dynamic interactions and underlying biological mechanisms in the prostate tumor microenvironment are yet to be available. In this work, we report the development of an *in vitro* human Prostate-Cancer-on-Chip model (PCoC) along with results pertaining to the interaction between the tumor and stroma within the established co-culture microenvironment.

## 2 Materials and methods

### 2.1 Cell lines and reagents

Immortalized primary human prostate basal epithelial cells (PrECs) were used as the normal epithelium in the prostate gland model (Berger et al., 2014). Benign human prostate fibroblasts (BHPrS1s) from Simon Hayward were used as normal stromal fibroblasts (Hayward et al., 1998). Three types of tumor cell lines, EMP, C4-2 and 22Rv1, were utilized in this work. EMP cells were generated by stably introducing Myc (M), shPten (P), and ΔErg (E) into PrECs, as previously described [Berger et al., 2014]. The PrECs and EMP cells were maintained in Keratinocyte Serum-Free Medium (KSFM, Gibco 17005042) supplemented with bovine pituitary extract (BPE), epidermal growth factor (EGF), and 1% penicillin-streptomycin. BHPrS1 cells were grown in RPMI 1640 medium (Gibco) with 5% Fetal Bovine Serum (FBS) and 1% penicillin-streptomycin. C4-2 and 22Rv1cells were obtained from ATCC and grown in RPMI 1640 medium (Gibco) with 5% FBS, 1% penicillin-streptomycin. Human prostate cancer spheroids isolated from a patient were used in one experiment. The de-identified tissue was obtained from the tissue repository under a standard IRB protocol. For invasion experiments, fluorescently-labeled cells were obtained by lentiviral infection with plasmids containing either green fluorescent protein (GFP) or red fluorescence protein (RFP) cDNAs. Specifically, mCherry-labeled BHPrS1 cells, GFP-labeled tumor cells, either C4-2 or 22Rv1, and normal PrEC cells were utilized. All cultures were maintained at 37°C in 5% CO_2_ with humidity >90%.

### 2.2 Prostate-cancer-on-chip (PCoC) model and microfluidic device design and fabrication

In normal prostate, stromal and epithelial layers are separated by a basement membrane rich in laminin and collagen. However, in prostate cancer, stromal cells are converted into cancer-associated fibroblasts (CAFs), which were observed at a higher density, differentially exposed to a multitude of tumor secreted factors leading to their heterogeneity within the tumor environment (Raskov et al., 2021). The basement membrane breaks down, and cancer cells infiltrate and invade the surrounding stroma. Thus, the prostate tumor is integrated into its stromal microenvironment resulting in physical cellular interactions during cancer progression.

**FIGURE 1:**
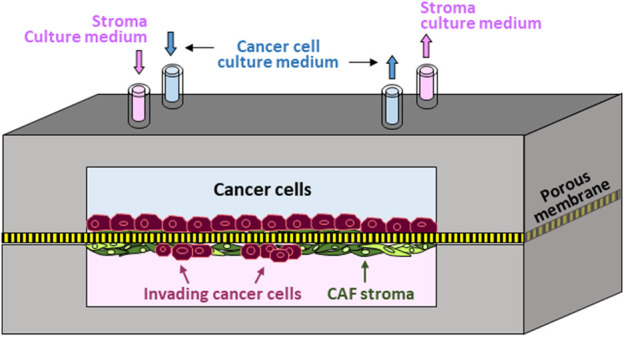
Prostate-Cancer-on-Chip conceptual model: Cancer and stromal fibroblast cells are co-cultured on opposite surfaces of a porous membrane separating the two aligned channels; normal fibroblasts conversion into CAFs by cancer cells is mediated by secreted soluble factors; large pore-size membrane allows reciprocal cell invasion into neighboring channels.

Accordingly, we established the first *in vitro* human prostate-cancer-on-chip (PCoC) model to incorporate prostate cancer cells and normal prostate stroma allowing them to interact. The model conceptual design (Figure 1) illustrates co-culturing prostate cancer cells and stromal fibroblasts in a pair of channels in a device. The channels are aligned on top of each other and separated by a porous membrane. Cancer cells and fibroblasts are plated on opposite surfaces of the separation membrane, establishing two distinct cultures of tumor and stroma. Membranes with smaller 0.8 µm-pores are large enough to allow exchange of biomolecules only across the membrane, due to diffusion, thereby establishing continuous cross-communication between the two cultures. Membranes with larger pore size of 8 µm permit biomolecule exchange leading to spatial concentration gradients. In addition, 8 µm-pore membrane also allows infiltrating cancer cells, and/or stromal cells, to cross the separation membrane invading into the neighboring culture. Steady and independent flow of culture medium through each channel is controlled by a syringe pump to maintain the co-culture providing continuous nutrient replenishment and waste removal.

**FIGURE 2:**
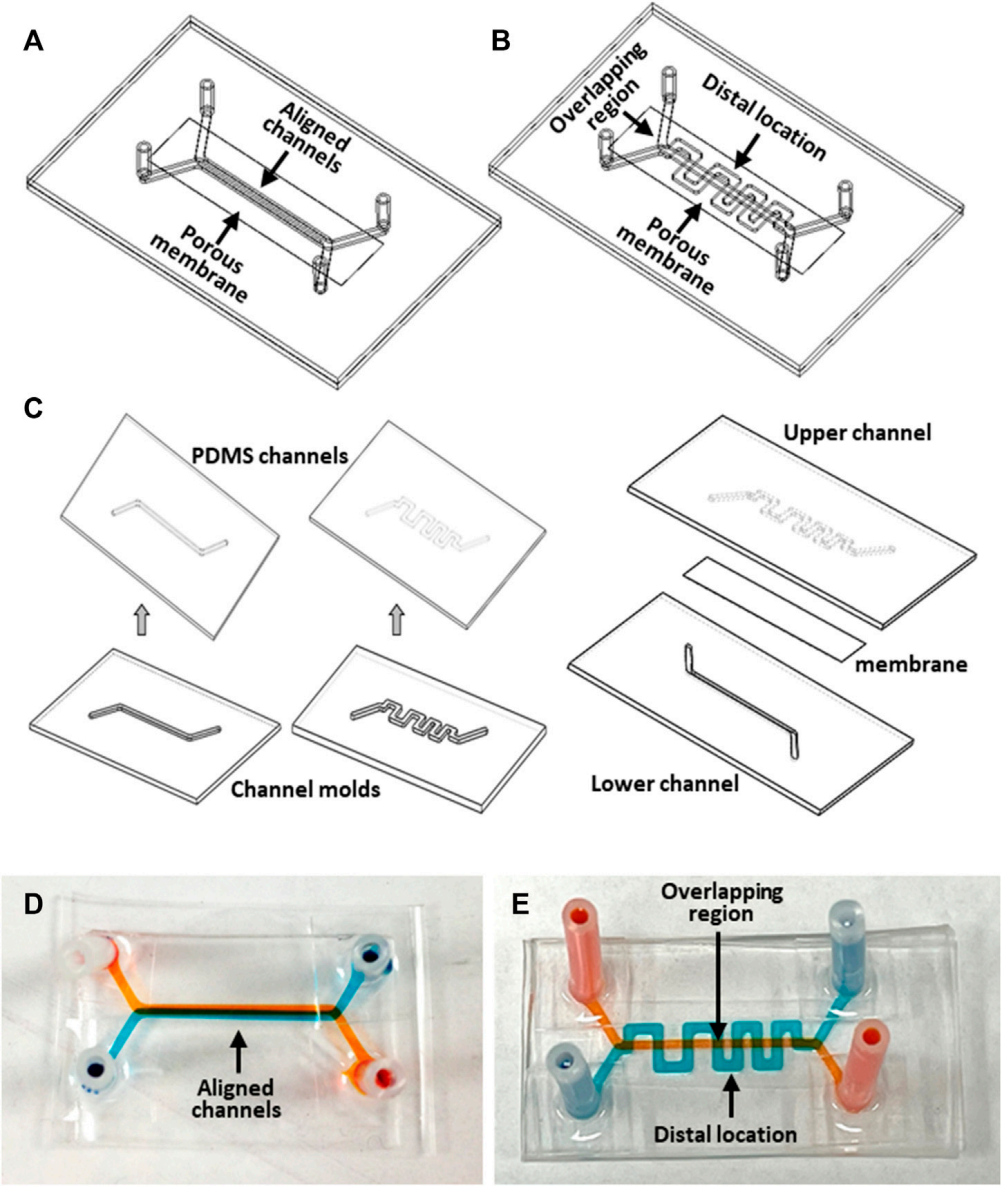
Microfluidic device fabrication: **(A, B)** Devices with aligned straight-straight channels and straight-serpentine channels, respectively; **(C)** major fabrication steps; **(D, E)** pictures of fabricated devices with straight-straight channels and with straight-serpentine channels, respectively. The channels were filled with different color dyes for visualization.

Two configurations of microfluidic devices were designed and fabricated. The first device design featured a face-to-face assembly of two straight channels separated by a porous membrane (Figure 2A). The porous membrane (ipCELLCULTURETM Track Etched Membrane, it4ip S.A., Belgium) is made of polyester with a thickness about 20 µm. The pore size of the separation membrane was selected to be either 0.8 µm or 8 µm with a porosity of 0.8% or 2%, respectively, depending on the specific experimental objective. Each of the two channels was 34 mm long, 1 mm wide, and 500 µm high, with the overlapping segment of the two straight channels about 20 mm in length. Inlet and outlet tubing adapters allowed device connection to an external fluid handling system. The second device design also featured two channels separated by a porous membrane; however, while one straight channel was retained as in the previous design, the configuration of the other 59 mm-long channel was modified to a serpentine shape with the same width and height (Figure 2B). The two channels had 8 selective overlapping regions. The serpentine channel consists of 16 orthogonal channel segments, perpendicular to the main channel axis, extending in both directions from the centerline axis of the straight channel to distal locations at a distance of 2.5 mm. This unique device design with 8 µm-pore membrane allows establishment of desired planar spatial concentration gradients along the orthogonal channel segments. It also enabled investigation of the spatially dependent interaction between the two cultures, such as cell invasion, migration and interaction. d tumor cell activities for microscopic observation.

The devices were made of optically transparent polydimethylsiloxane (PDMS, Sylgard 184, Dow Corning Corporation) utilizing soft lithography technology (Jiang et al., 2019). A mold for each microchannel was fabricated in an aluminum block using a computer numerical control machine (CNC) based on a 3D CAD design. Fabrication of the devices involved the following major steps (Figure 2C). PDMS, prepared by mixing base elastomer and curing agent at a 10:1 w/w ratio, was dispensed on a mold. After removal of air bubbles form the PDMS under vacuum, the mold with the PDMS was placed on a leveled surface at room temperature until the PDMS was fully cured. The PDMS layer was then peeled off from the mold to obtain microchannel replicates. A porous membrane was treated for 1-min in an oxygen-plasma reactor (Harrick Plasma, United States), then immediately immersed in 5% 3-Aminopropyltriethoxysilane (APTES, Sigma-Aldrich) solution in water, followed by incubation at 50°C for about 30min to prepare its surfaces for bonding. The bonding surfaces of a pair of PDMS channel replicates, with inlet and outlet holes, were activated by oxygen plasma treatment. The two PDMS channels were aligned and bonded together with dried membrane sandwiched between them. The assembled device was incubated at 50°C for 30min to enhance the bonding adhesion. Finally, inlet and outlet adapter tubing were attached to the channel inlet and outlet holes for connection with the external flow control system. Pictures of the fabricated straight and serpentine devices filled with different color dyes depict the pair of two individual channels in a device (Figure 2D, E).

### 2.3 Experimental procedures

All experiments started with channel surface coating to enhance cell adhesion and provide physiologically relevant matrix for cell cultures. Tumor channels were coated with 10 μg/mL laminin 1 (ThermoFisher, 1.2 mg/mL) in 1×PBS w/Ca/Mg. Fibroblast channels were coated using either a solution of 20 μg/mL fibronectin (Sigma F0895) in 1×PBS w/o Ca/Mg, or a solution containing 500 μg/mL rat tail collagen I (Corning 354236) with 20 μg/mL fibronectin (Sigma F0895) and 62.5 μg/mL Ethylene glycol-bis (succinic acid N-hydroxysuccinimide ester) (PEG) (Sigma E3257) in 1×PBS w/o Ca/Mg. The solution pH was adjusted to be 7.0 incrementally with 0.1M NaOH. Prior to a coating process, the channels were first washed using 70% ETOH several times and sterilized for an hour under UV irradiation inside a biosafety cabinet at room temperature, followed by washing several times with 1×PBS w/o Ca/Mg. Each channel was then filled with its designated coating solution, and the device was placed inside the biosafety cabinet under UV for 1 hour at room temperature. The coating solution was next removed from the channel, retaining a coated thin film on its inner surface, and the device was placed in an incubator for 1 hour at 37°C to cure the film. Coating a channel pair in a single device followed a similar process conducted either in sequence or in parallel. Upon completion of the coating procedure, the channels were washed with 1×PBS and the culture medium. Devices with the corresponding culture medium in the channels were incubated for more than 30min in final preparation for cell loading.

In a typical co-culture experiment, stromal fibroblast cell suspension was first flowed into the upper channel using a pipette. The device was placed overnight in an incubator at 37°C to ensure firm fibroblasts attachment to the membrane upper side. Then, cancer cell suspension was flowed into the lower channel, and the device was immediately inverted allowing cell attachment to the membrane lower surface overnight in the incubator. Suspensions of 2×10^6^ cells/mL of either cell type were used for initial cell seeding to obtain fully confluent layers. In general, either type of cells could be cultured in the upper or lower channel. In one case, both cell types were layered on the membrane in the same channel. Once cell attachment to membrane surfaces was confirmed, culture medium was manually driven through each channel slowly to wash away loose cells inside the channels. Attached cell layers in both channels remained on the membrane after the manual wash at a rate about 100 μL/min, corresponding to a wall shear stress of 0.14 dynes/cm^2^. The device was then connected to the external fluid handling system. During all co-culture experiments, cell culture was maintained under flow of culture medium through its corresponding channel at a constant rate of 30 μL/h controlled by a syringe pump. The corresponding wall shear stress under the constant culture medium flow is estimated to be 1.4 × 10^−3^ dynes/cm^2^. For synthetic androgen treatment, R1881 was administered in the flow to the stromal culture only, upstream of its channel inlet, at a concentration of 50 nM every other day (Jiang et al., 2019).

For the primary tumor spheroid experiment, spheroids about of 20µm–100 µm in size were suspended in 1:1 culture medium and Matrigel (Corning^®^), injected into the upper channel, and cured at 37°C for 30min. Spheroid cultures were maintained in devices for 10 days with medium flow in the lower empty channel while replenishing the culture medium daily at the upper channel inlet/outlet tubing adaptors. Them stromal BHPrS1 cells were plated on the membrane in the lower channel to establish a co-culture of tumor spheroids with BHPrS1 cells. The co-culture lasted for 10 days, under constant culture medium flow of 30 μL/h through the BHPrS1 channel, while culture medium for the tumor spheroid channel was manually replenished daily.

### 2.4 Immunofluorescence

Antibodies were used to detect biomarkers in cells at the completion of experiments, and information regarding the utilized antibodies is summarized in Table 1. Immune-staining processes were performed by manually flowing proper solutions through the channels while the cells were attached to both surfaces of the separation membrane. Briefly, the cells were washed three times with 1×PBS and then fixed with 4% paraformaldehyde for 10min at room temperature. After three washes with 1×PBS, cells were permeabilized in 0.2% Triton in 1×PBS for 5 min, followed by four washes with 1× PBS. Either goat serum (5% in 1× PBS), donkey serum (5% in 1× PBS), or BSA (1% in 1× PBS) was used for 1-h blocking at room temperature. Cells were then incubated overnight at 4°C in a selected primary-antibody solution diluted in the blocking solution at a ratio of 1:100. After four washes with 1×PBS, cells were incubated in a complimentary secondary-antibody solution in 1×PBS (1:500) for 1 hour at room temperature. Cells were washed thoroughly using 1×PBS. Immunofluorescence in cells was measured based on the signal of the markers using a Nikon Eclipse TE2000-U microscope under various objectives (×4, 10×, 20×). Images under higher magnifications (×40, and 100×) were also acquired after de-bonding the devices to remove the separation membranes with cells attached. Each membrane was mounted in 70%–90% glycerol solution and sandwiched between two Number 1.5 glass coverslips. The stained samples were kept in dark in a humid box at 4°C.

**TABLE 1:** Antibodies for immunofluorescence study.

Antibody	Host species	Company (CAT #)
AR (441)	mouse rabbit	Santa Cruz (sc-7305) Cell signaling technology (5,153)
TMPRSS2	mouse	University of Washington (P5H9-A3)
HMWCK	mouse	Dako (M0630)
αSMA	mouse	Sigma/Millipore (A2547)
COL1A1	rabbit	Genetex (GTX112731)
FAP	rabbit	Genetex (GTX102732)
POSTN	rabbit	Santa Cruz (sc-398631)
Vimentin	goat	Sigma/Millipore (V4630)
Secondary antibody 488	goat anti-mouse	Thermofisher (A 32723)
Secondary antibody 555	goat anti-mouse	Thermofisher (A 32727)
Secondary antibody 488	goat anti-rabbit	Thermofisher (A 32731)
Secondary antibody 555	goat anti-rabbit	Thermofisher (A 32732)
Secondary antibody 647	goat anti-rabbit	Thermofisher (A 32733)
Secondary antibody 647	donkey anti-goat	Thermofisher (A 32849)

### 2.5 Imaging data collection and analysis

At least 2-4 devices were used in a typical set of experiments serving as duplicates as well as controls for comparison analysis, while every experiment was performed at least 2× for repeatability check. Through the duration of an experiment, cell activities in both channels of each device were scanned and inspected at least once every 2 days, and more than 20 representative live-cell images under various magnifications were recorded at different locations along the channels to monitor changes at each inspection day. The distance traveled by the farthest migrating cancer cell in the invaded channel was first estimated through the microscope eyepiece after examining all 16 orthogonal channel segments of the device, and then measured on the recorded images using ImageJ. Both average and standard deviation (SD) of the farthest migrating-cell distance were obtained from at least 4 different devices of each experiment. For immunofluorescence imaging, fluorescent signals in cells throughout the devices were first examined through the microscope eyepiece, and more than 20 images at different locations along a device were acquired under adequate magnifications. Representative images were selected for the result discussion.

### 2.6 Cytokine analysis

Invitrogen Cytokine ELISA kits for PDGF AB, TGFβ, and TNFα were purchased from ThermoFisher. Conditioned media from each channel was assessed using the protocols supplied with the kit alongside a set of standards. Raw reads after measuring signal from 450 nm wavelength were extrapolated using the standard curve to yield the cytokine concentration in pg/µL unit for each sample. Reads from 4 samples each were averaged and plotted. Statistical significance at each time point was determined using standard student T-test relative to the earliest time point of Day 7, and *p* < 0.05 was considered significant.

## 3 Results and discussions

### 3.1 Numerical simulations of convection-diffusion flow in the device co-culture

Tumor-stroma communication in the PCoC is mediated via signaling molecules secreted by both cell populations. Secreted soluble factors, such as proteins, are released into the extracellular domain where, while convected downstream, also diffuse within both channels and across the membrane due to concentration gradients. Consequently, signaling molecules transported through the membrane pores can reach neighboring cells and bind to their receptors initiating physiological responses. This bi-directional tumor-stroma communication depends on the signal strength, which is a direct function of the signaling-molecule concentration in the vicinity of receiving cells. Low flowrate results in strong signals but severe culture medium mixing between the two channels, which is detrimental to both cells cultures. Conversely, high flowrate restricts media mixing but yields poor cell signaling. It is critical therefore to fine-tune the convection-diffusion balance for the creation of an adequate microenvironment supporting effective cell signaling between the two cultures while also maintaining the individual tumor and stroma cultures. Peclet number, *Pe*, is the control parameter characterizing the relative contributions of these two mass transport physical mechanisms defined as:

$$Pe=\frac{LU}{D}$$ (1)

where, *L* and *U* are characteristic length and velocity scales, respectively, and *D* is the diffusion coefficient. In general, *Pe* > 1 indicates dominant convection, while *Pe* < 1 indicates dominant diffusion. In our model system, the aim is to attain dominant diffusion near the membrane and cell layers, i.e., *Pe*<<1, with limited convection. Away from each cell layer, on the other hand, the local flow velocity should be high enough such that for convection to be dominant relative to diffusion, i.e., *Pe*>>1. The diffusion coefficient in the culture medium is estimated to be on the order of 10^–10^ m^2^/s (Savinell et al., 1989). Thus, the local flow velocity must be in the range of 1–10 μm/s to maintain dominant convection.

**FIGURE 3:**
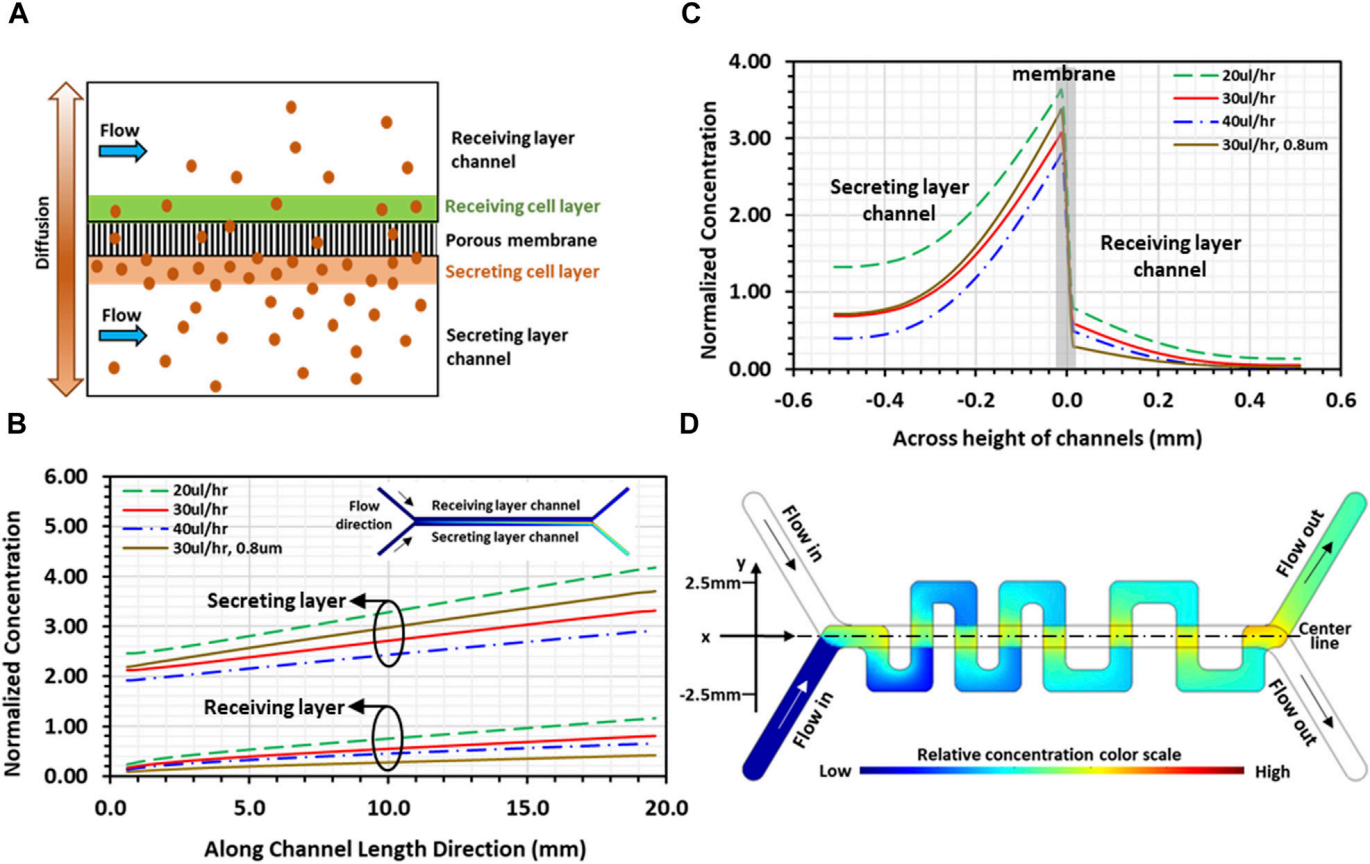
Simulations of the convection-diffusion flow in a microfluidic device: **(A)** Schematic illustration of 2D physical model used for simulations depicting transport of signaling molecules through porous membrane due to diffusion from the secreting layer to the receiving layer channel; **(B)** 2D simulation result of streamwise normalized concentration profiles of secreted molecules along both channels under various flowrates, at a distance 40 µm away from membrane, with the corresponding rainbow maps of planar concentration distributions (inset; blue and red colors represent low and high concentrations, respectively). **(C)** 2D simulation result of cross-stream normalized concentration profiles of secreted molecules, at mid-distance between channel inlet and outlet, under various flowrates; and **(D)** 3D simulation result of planar concentration rainbow map showing normalized spatial concentration distribution in serpentine channel. All results are for a device with 8 µm-pore membrane, except curves in brown color in **(B, C)**, which are for a device with 0.8um-pore membrane at flow rate of 30 μL/h.

Numerical computations were conducted using COMSOL to gain insight into molecules transport interaction in the co-culture. A simplified 2D physical model featuring two identical straight channels aligned and separated by porous membrane was adopted for the simulations to highlight the convective-diffusive transport of signaling molecules (Figure 3A). Each straight channel was 20 mm long and 500 µm high, while the pore size of the 20 µm-thick separation membrane was 8 µm with 2% porosity. As indicated, the communication between the 2 cell layers in the co-culture is bi-directional; namely, each cell layer is simultaneously secreting and receiving signals. However, for illustration purposes, a cell culture on one membrane surface was selected as the secreting layer with the other cell culture on the opposite membrane surface as the receiving layer; physical thicknesses of both cell layers were considered zero in the model. The Effect of PDMS absorption of molecules should be considered for transient flow analysis before reaching steady state. In the present system, cells continuously secret molecules that were retained in proximity of the 2 cell layers attached on the membrane. Under steady-state condition, the concentration distribution in the system reached an equilibrium, whereby all flow properties including solute concentrations are constant in time. Consequently, after the initial absorption of molecules, additional adsorption by the PDMS channel walls and polyester membrane is zero for steady flow. In both channel domains, including the inlet/outlet segments of the channels, steady-state incompressible Naiver-Stokes equations coupled with the convection-diffusion equation were solved using water properties at 37°C, whereas only diffusion with zero velocity was considered in the porous membrane domain. In the flow module, no-slip boundary condition was applied to all channel walls as well as on the surfaces of the membrane (or cell layers). A uniform flow velocity was set at the inlet of each channel, and zero gage-pressure was set at the outlet of each channel. In the convection-diffusion module, zero concentration boundary condition and convective flux were applied at the inlet and outlet of each channel, respectively. A constant concentration flux was applied at the secreting cell layer (Francis and Palsson, 1997), whereas insulation boundary condition was imposed on all channel walls. A diffusion coefficient of 0.5 × 10^−10^ m^2^/s was selected for the medium flow in the two channels, whereas the diffusion coefficient in the membrane domain was 1 × 10^−12^ m^2^/s for the 8 µm-pore (2% porosity) membrane. Continuity in concentration at the interface between the membrane domain and the receiving channel flow domain was applied. Under these conditions, convection away from the cell layer is indeed dominant, *Pe*>>1, confining the diffusion to the regions in close proximity of the cell layers. Streamwise normalized concentration profiles of secreted molecules along the channels, from inlet to outlet at a distance 40 µm away from both membrane surfaces, are presented for 20, 30, and 40 μL/h flow rates (Figure 3B). All concentrations monotonically increase along the flow direction attributed to the balance between stream-wise convection and cross-stream diffusion of secreted molecules. Normalized concentration profiles of the receiving cell layer show similar trends, i.e., increasing linearly along the flow direction, suggesting that the receiving layer microenvironment near the channel outlet contains significantly higher concentration of the secreted molecules than the microenvironment near the channel inlet. Furthermore, at the same stream-wise location, the concentration in the receiving channel is systematically about 20% of the secreting channel concentration. As expected, increasing flow rate leads to reduced concentrations in both secreting and receiving channels. Rainbow color concentration maps depict the relative concentrations in the two channels (inset of Figure 3B), where blue color represents the lowest and red the highest concentration. Cross-stream normalized concentration profiles, at half distance between the channels inlets and outlets, are presented for the same flow rates (Figure 3C). The results show strong concentration gradients across the cell layers on each side of the membrane, which is evident due to the low effective diffusion coefficient (Young et al., 1980). In the receiving layer channel, concentration is high at the cell layer on the membrane, but rapidly diminishes at locations father away from the cell layer, suggesting that signaling molecules are well confined to the vicinity of the receiving cell layer. Similarly, in the secreting channel, the concentrations decrease from their peaks at the secreting cell layer with increasing distance away from the membrane. The simulation was repeated for the 0.8 µm-pore membrane device with a lower diffusion coefficient 0.4 × 10^−12^ m^2^/s (porosity 0.8%). The results (brown curves in Figures 3B, C) show similar concentration profiles in both streamwise and cross-stream directions with enhanced gradients across the cell layers, as expected. Notably, the diminishing concentration away from the receiving cell layer indicates a limited diffusion range at a reduced concentration level.

To obtain spatial concentration distributions in the device designed with straight and serpentine channels, 3D numerical simulations were performed to solve the steady-state coupled incompressible Naiver-Stokes and convection-diffusion equations only in the serpentine channel domain, i.e., the receiving cell layer channel. In the flow module, a constant volumetric flow rate was set at the inlet of the channel and zero gage-pressure was set at the outlet of the channel. The difference in channel length, 34mm and 59 mm for the straight and the serpentine channel, respectively, resulted in a pressure difference across the membrane between the two channel flows at the overlapping regions. This pressure difference, estimated to be on the order of 10^–3^ Pa at a flow rate of 30 μL/h, was too low to drive the flow across the membrane through 8 µm-pores; hence, there was no cross-flow through the porous membrane, and no-slip boundary condition was applied at the channel walls as well as at the zero-thickness cell layer surface. In the convection-diffusion module, the same diffusion coefficient for the medium flow in the 2D simulation was selected. Zero concentration and convective flux were applied at the channel inlet and outlet, respectively, and insulation boundary condition was applied at the channel walls. On the receiving cell-layer, a concentration function was applied only at the overlapping regions between the serpentine and the straight channels. The function was determined based on the concentration distribution of the receiving layer obtained in the 2D simulations (Figure 3B), which increases linearly with distance along the flow direction in the straight channel. The results demonstrate that concentration of soluble factors in the serpentine channel is higher near the overlapping regions and lower at distal locations farther from the overlapping regions, establishing spatial concentration gradients in all orthogonal channel segments (Figure 3D). Consequently, tumor-stroma signaling is abundant close to the overlapping regions, driving tumor-stroma interaction, whereas the signaling is weaker at distal locations of the serpentine channel. These numerical simulations served as a primary guidance in determining the experimental conditions required for long-term maintenance of cell co-cultures while allowing continuous communication between the two cultures in our PCoC model.

### 3.2 Tumor cells conversion of normal fibroblasts into CAFs mediated by diffused factors

The first set of experiments was designed to investigate the effect of tumor cells on the neighboring normal stromal fibroblasts with no physical contact between the two types of cells. BHPrS1 fibroblasts were co-cultured with either oncogene-induced human tumor cells (EMP), metastatic C4-2 tumor cells, or primary tumor cells isolated from a patient, in devices with 0.8 µm-pore membranes. In these co-cultures, solutes originated from each culture transported to the adjacent culture establishing communications between the tumor and fibroblast cells with no physical contact between the 2 cell types. Co-cultures were maintained for about 15 days under steady culture medium flow of 30 μL/h in each channel. R1881 treatment was administered every other day only into the fibroblast culture medium under flow at a concentration of 50 nM. Control experiments were conducted under the same conditions but with normal PrECs replacing tumor cells. Immunostaining of biomarkers specific for tumor cells and CAFs was performed, at the completion of each experiment, followed by immunofluorescence microscopic imaging.

**FIGURE 4:**
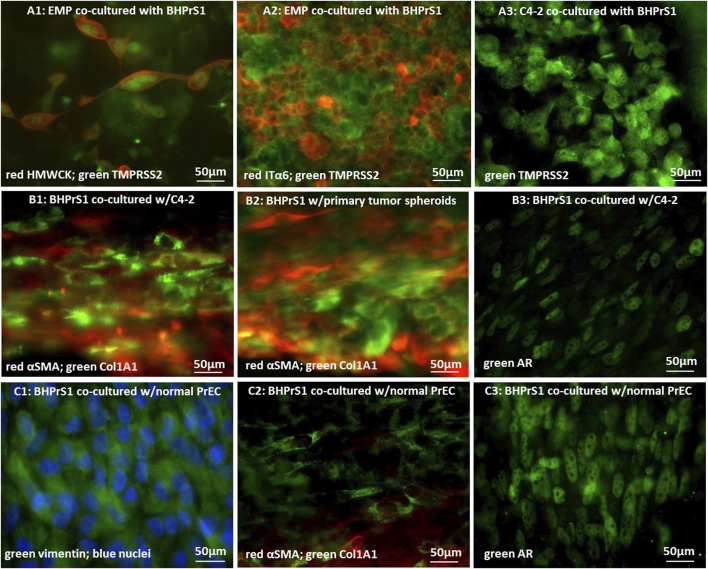
Immunofluorescence microscopic images of cancer cells and stromal fibroblasts co-cultured in 0.8 µm-pore membrane devices: **(A1)** EMP cells stained for red-HMWCK and green-TMPRSS2, or **(A2)** red-ITGα6 and green-TMPRSS2, and **(A3)** C4-2 cells stained for green-TMPRSS2; **(B1)** BHPrS1 cells co-cultured with C4-2 cells and stained for red-αSMA and green-COLlA1, **(B2)** BHPrS1 cells co-cultured with primary tumor spheroids and stained for red-αSMA and green-COLlA1, and **(B3)** BHPrS1 cells co-cultured with C4-2 cells and stained for green-AR; **(C1)** BHPrS1 cells co-cultured with normal PrECs and stained for green-vimentin and blue-nuclei; **(C2)** green COL1A1 and red-αSMA, and **(C3)** green-AR.

Several markers were selected to examine the prostate cancer cells in the co-cultures. TMPRSS2 is known to be highly expressed in metastatic prostate cancer cells, the activity of which was reported to regulate and promote prostate cancer cell invasion and metastasis (Lucas et al., 2014; Ko et al., 2015). High molecular weight cytokeratin, HMWCK, has been suggested as a marker for distinguishing malignant from benign prostatic epithelial structures because they selectively label basal cells that are missing in tumors (Kahane et al., 1995). Other studies however reported that HMWCK is expressed in prostate adenocarcinoma, particularly in invasive and metastatic tumors (Yang et al., 1999; Parker et al., 2003; Kunju et al., 2006; Multhaupt et al., 2000; Lu et al., 2023). Integrin α6, ITGα6, has been associated with migratory and invasive phenotype in human prostate cancer. A considerable amount of work has implied that ITGα6 plays an important role in tumor cell migration and modification of the tumor environment (Rabinovotz et al., 1995; King et al., 2008; Goel et al., 2008; Rubenstein et al., 2019). In our experiments, the cancer marker TMPRSS2 is indeed overexpressed in the entire population of tumor cells, either EMP or C4-2, confirming their prostate cancer identity (Figures 4A1–4A3). A few EMP cells also expressed basal HMWCK (Figure 4A1), while a considerable number of tumor cells expressed ITGα6 (Figure 4A2). These results confirm that the tumor cells in the PCoC model retain known markers of prostate tumor cells.

Stromal fibroblasts in the co-cultures were next evaluated using a couple of antibody markers. Expression of alpha smooth muscle actin (αSMA) was reported to be very low or heterogeneous with occasional expression in individual stromal cells, whereas the level of αSMA expression was significantly higher in CAFs (Storch et al., 2007; Castelló-Cros et al., 2009; Bharath Rao et al., 2014; Parikh et al., 2014; Rao et al., 2014; Angaravati et al., 2017; Gillard et al., 2018). Furthermore, during tumor progression, CAFs actively modify the ECM as characterized by collagen degrading, re-depositing, and cross-linking. COL1A1 expression is increased in CAFs, and its signaling promotes cancer invasion (Hall et al., 2008; Fang et al., 2014). Accordingly, the fibroblasts in the devices were immuno-stained with αSMA and COL1A1 antibodies. Stromal fibroblasts co-cultured with tumor C4-2 cells strongly expressed the two CAF markers, αSMA and COL1A1 (Figure 4B1). The αSMA expression was distributed throughout the cell cytoplasm. Interestingly, cells expressing αSMA and those expressing COL1A1 often appeared as two distinct sub-populations, confirming the known heterogeneity within CAF populations. In addition, spheroids generated from a fresh primary prostate tumor of a patient were co-cultured with normal BHPrS1 fibroblasts in the device model. Tumor spheroids also converted normal stromal fibroblasts to CAFs, with high expression of αSMA and COL1A1 (Figure 4B2). Similarly, CAFs did not generally co-express αSMA and COL1A1, but appeared to be divided into two distinct subgroups; one group expressed αSMA while the other expressed COL1A1. In control experiments of co-culturing BHPrS1 with normal PrECs, BHPrS1 cells, with a robust expression of vimentine (Figure 4C1), showed very weak expressions of αSMA and COL1A1 (Figure 4C2). The AR expression in BHPrS1 cells co-cultured with normal PrECs was very high and localized in cell nuclei (Figure 4C3). In comparison, AR expression in CAFs was dramatically decreased (Figure 4B3).

In summary, exposure of stromal fibroblasts to cancer cells, without physical contact, results in increased expression of cancer-associated fibroblast (CAF) markers αSMA and COL1A1. This indicates that cell signaling by secreted factors from cancer cells into their tumor microenvironment, across the separation membrane, led to stromal fibroblast conversion. The expression of the Androgen Receptor (AR) in stromal fibroblasts, necessary for maintaining normal epithelial differentiation, was found to be lost in correlation with higher Gleason Grade and poor outcome (Hahn et al., 2023). The distinguishable decrease in AR expression in CAFs is consistent with observations in human prostate cancer patient samples (Henshall et al., 2001; Wikström et al., 2009; Franco et al., 2010). In our previous normal Prostate-on-Chip model, secreted morphogens induced by androgen receptor (AR) signaling in the stromal fibroblasts were found to promote PrEC differentiation (Jiang et al., 2019). Hence, the reduction of AR in stromal fibroblasts exposed to cancer cells unfavorably affects normal epithelial differentiation and homeostasis, which has a negative impact on maintaining healthy prostate functions.

### 3.3 Tumor-stroma interaction dependence on local concentration of signaling molecules

The second set of experiments was designed to investigate the signaling strength effect, proportional to local concentration of tumor-secreted factors, on stromal fibroblasts conversion to CAFs within the co-culture. Devices consisting of a straight channel for the tumor cells and a serpentine channel for the stromal fibroblasts with 8 µm-pore membrane were used. In this configuration, local high and low concentrations of tumor-secreted factors are respectively located in the aligned and distal regions of the fibroblast serpentine channel (Figure 3D). Stromal BHPrS1 fibroblasts were co-cultured with metastatic tumor cell lines, either C4-2 or 22Rv1, in devices with 8 µm-pore membranes. The 2 cell types can communicate through solutes in the culture medium as well as via physical contact, i.e., each type of cells can invade into their neighboring channel. To clearly monitor cell invasion, cancer cells were tagged with green fluorescent protein (GFP). Co-cultures were maintained for 12–17 days under continuous flowrate of 30 μL/h in each channel. R1881 treatment was administered as described in Section 2.3. Control experiments were performed under the same conditions but with normal PrECs replacing the tumor cells. Once an experiment was completed, immunostaining was performed using markers specific for tumor cells and CAFs and followed by fluorescence imaging.

**FIGURE 5:**
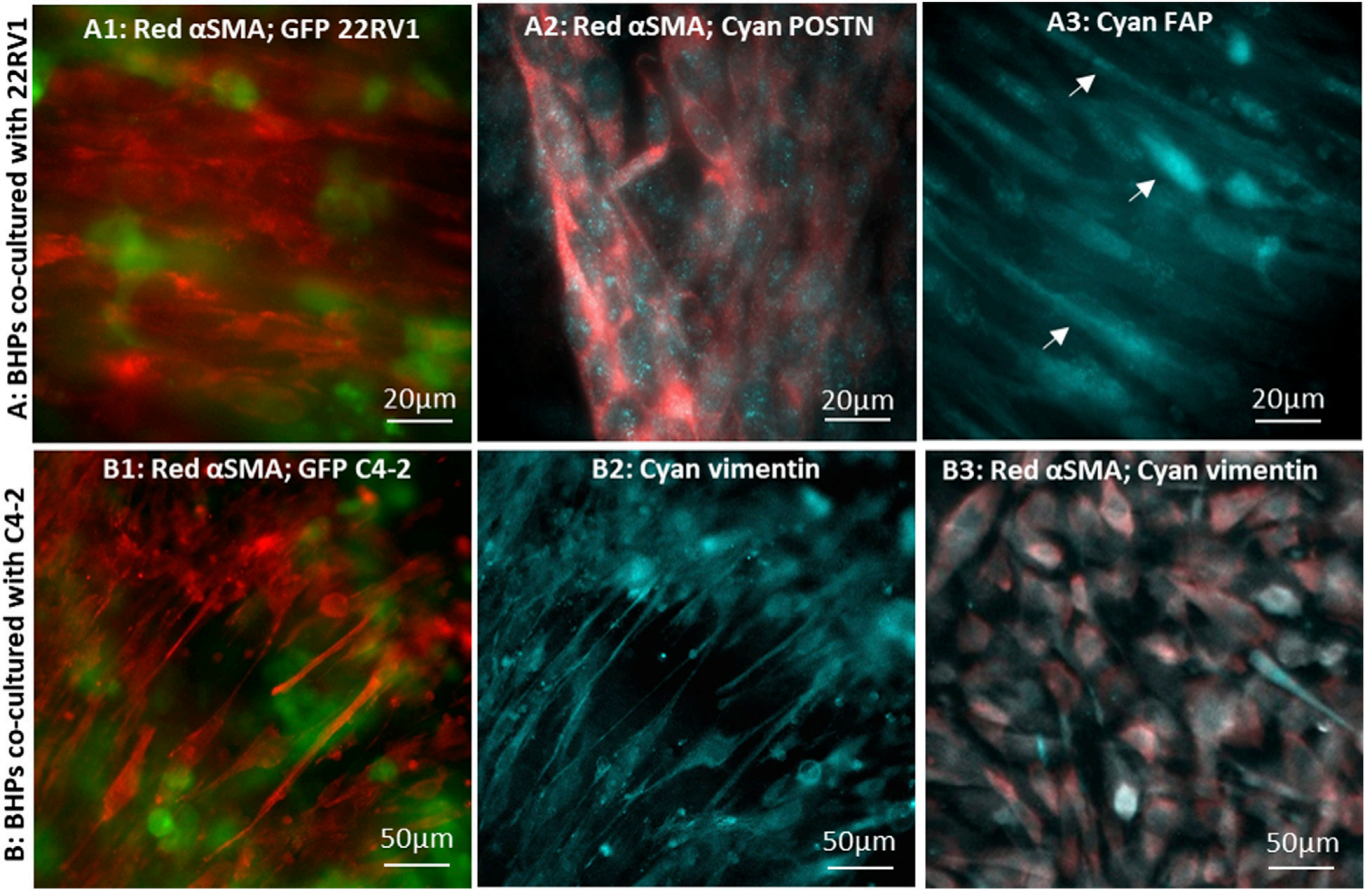
Immunofluorescence microscopic images of CAFs converted by cancer cells co-cultured in 8 µm-pore membrane devices: BHPrS1 cells in close proximity with cancer GFP-22Rv1 cells stained for **(A1)** red-αSMA, **(A2)** red-αSMA and cyan-POSTN, and **(A3)** cyan-FAP. BHPrS1 cells in close proximity with cancer GFP-C4-2 cells stained for **(B1)** red-αSMA, **(B2)** cyan-vimentin; **(B3)** BHPrS1 cells at a distal location in the serpentine channel far away from C4-2 cancer cells stained for red-αSMA and cyan-vimentin.

CAF markers in stromal fibroblasts, co-cultured with tumor cells in devices with 8 µm-pore membranes, were examined. Similar to the results obtained in devices with 0.8 µm-pore membranes (Figure 4), stromal fibroblasts in close proximity to the cancer cells, either 22Rv1 or C4-2, displayed higher αSMA expression (Figures 5A1, A2; Figure 5B1), confirming their conversion into CAFs induced by the tumor cells. Furthermore, it was noted that the CAFs assumed highly elongated spindled-shaped morphology and aligned tightly in bundles, consistent with reports suggesting that CAFs grow in a highly aligned organization with most fibroblasts orientated in the same direction (Clark et al., 2013). Expression of αSMA was detected in the cytoplasm of most CAFs (Figures 5A1, A2), whereas αSMA-containing long filamentous fibers were observed in a subset of stromal fibroblasts (Figure 5B1), further displaying the heterogeneous nature in the CAF population.

Periostin (POSTN) has been recognized as a secretory extracellular matrix protein associated with tumor progression and shorter survival in prostate cancer (González-González and Alonso, 2018; Cattrini et al., 2020; Dorafshan et al., 2022), and is upregulated in the stroma of high grade/stage prostate cancer (Tischer et al., 2010; Nuzzo et al., 2012; Tian et al., 2015; Tsunoda et al., 2009). In CAFs co-cultured with 22Rv1 cancer cells, POSTN expression pattern appears as finely sprinkled distribution in the cytoplasm with higher expression level at locations corresponding to the Golgi (Figure 5A2), consistent with other reported findings (Qin et al., 2016; Yoshioka et al., 2002; Kii et al., 2016). We also examined fibroblast activation protein (FAP) marker, which was found to be expressed in the stroma of human prostate cancer tissues (Tuxhorn et al., 2002; Orimo and Weinberg, 2007; Nurmik et al., 2020). FAP signal was detected in CAFs with highly elongated morphology (Figure 5A3); however, the FAP signal was dim and indistinct in fibroblasts without the elongated morphology. As anticipated, αSMA-expressing CAFs were observed predominately at regions close to tumor cells in co-culture with C4-2 cells; these cells also co-expressed vimentin (Figures 5B1, B2) (Lang et al., 2002; Zhao et al., 2008). However, stromal fibroblasts, at distal locations in the serpentine channel (Figure 2E), had a barely detectable αSMA signal and failed to align (Figure 5B3). These observations demonstrate that stromal fibroblasts close to the tumor have converted into CAFs, while those far away from the tumor were either in a developing stage or remained un-affected normal fibroblasts (Castelló-Cros et al., 2009; Vermeulen et al., 2010).

The major difference between the CAF and non-CAF regions is the local concentration level due to spatial concentration gradients of tumor-secreted factors (Figure 3D). It is not clear whether the CAF conversion is an on-off switch mechanism requiring a threshold concentration level of tumor secreted factors for the process to occur, or it is a gradual and/or cumulative process independent of the local concentration level, whereby stromal fibroblasts would eventually be converted to CAFs after exposure to tumor environment for sufficient length of time. Nevertheless, fibroblasts in the overlapping regions exhibit distinct differences in morphology and CAF marker expression level than those at distal locations farther away from tumor cells. The results confirmed that normal fibroblasts conversion into CAFs is induced by tumor cells and, furthermore, revealed that tumor-stroma interaction depends on local signal strength stemming from spatial concentration gradients of secreted factors.

### 3.4 Invasion of tumor and fibroblasts in a reciprocal manner in 8 µm-pore membrane devices

Invasion of tumor cells into the stromal fibroblasts was next investigated using the devices with straight and serpentine channels separated by an 8 µm-pore membrane. BHPrS1 fibroblasts were co-cultured with two different metastatic prostate tumor cell lines, 22Rv1 or C4-2, where the cancer cells and stromal fibroblasts were allowed to communicate not only through solutes in the culture medium but also via physical contact through the pores. Establishing the co-cultures in the straight/serpentine device enabled continuous visual tracking of cancer cells invading the fibroblast channel through the separation membrane (Figure 1B, E). Invading cancer cells in the overlapping regions can migrate into the neighboring fibroblast channel in a direction perpendicular to their original tumor channel. Cells were tagged with green (GFP) or red (mCherry) markers to facilitate detection and monitoring of invasion activities of different-colored cells in a channel. The co-cultures were maintained for 17 days under continuous culture medium flow at 30 μL/h in each channel, R1881 treatment was administered as described in Section 2.3, and control experiments were performed with normal PrECs replacing the tumor cells.

**FIGURE 6:**
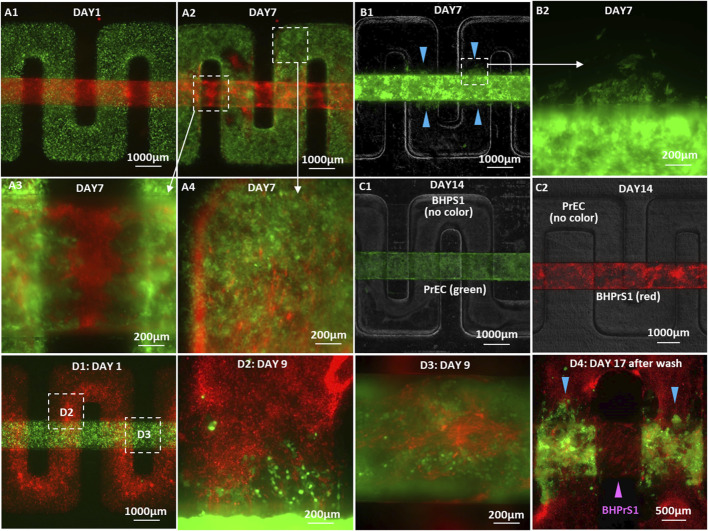
Microscopic images of cancer cell invasion in co-cultures with BHPrS1 fibroblasts: **(A1)** GFP-22Rv1 cancer cells plated on the membrane in the upper serpentine channel and mCherry-BHPrS1 fibroblasts plated on the membrane in the lower straight channel on Day 1, **(A2)** 22Rv1 cells detected in the stroma channel and BHPrS1 CAFs in the cancer channel on Day 7, **(A3)** high-magnification images of invading cancer cells in the stroma channel, and **(A4)** CAFs in the cancer cell channel on Day 7. GFP-22Rv1 cells plated on the membrane in the lower straight channel and un-labeled BHPrS1 cells plated on the membrane in the upper serpentine channel with invading 22Rv1 cells detected in the stroma channel on Day 7 at **(B1)** low and **(B2)** high magnifications. **(C1)** In control PrEC/BHPrS1 co-cultures, on Day 14, neither **(C1)** GFP-PrEC cells nor **(C2)** mCherry-BHPrS1 cells invade neighboring channel. **(D1)** GFP-C4-2 cells plated on the membrane in the lower straight channel and mCherry-BHPrS1 fibroblasts plated on the membrane in the upper serpentine channel on Day 1, **(D2)** invasion of C4-2 cells into the stroma channel and **(D3)** infiltration of BHPrS1 cells into the cancer cell channel on Day 9 at the respective locations indicated by white squares in **(D1)**, and **(D4)** retained C4-2 cancer cells attached to the fibroblasts in the stroma channel (blue color arrows) with infiltrated BHPrS1 fibroblasts attached to the tumor channel surfaces (magenta color arrow) after washing the channels with medium.

Co-cultures of tumor and stroma were established for the invasion experiments, where GFP-labeled 22Rv1 cancer cells were plated on the membrane in the upper serpentine channel and mCherry-labeled BHPrS1 fibroblasts plated on the membrane in the lower straight channel (Figure 6A1). On Day 7, 22Rv1 tumor cells were observed in the fibroblast channel (Figures 6A2, A3), and fibroblasts were also present in the cancer channel (Figure 6A4). The invading 22Rv1 cells, after penetrating through the separation membrane in the successive overlapping regions, migrated along the fibroblast channel while displacing the BHPrS1 cells (Figure 6A3). To rule out the role of gravity in cell invasion, the placement of the 2 cell types in the device was flipped; indeed, similar invasion patterns were observed regardless of the relative up/down positions of the two types of cells (Figures 6A1; Figure B1). 22Rv1 cells invaded into BHPrS1 channel when they were plated on the membrane in the lower (Figures 6A1–A4) or upper side of the membrane (Figures 6B1, B2). In contrast, normal PrECs co-cultured with BHPrS1 fibroblasts in devices with 8 µm-pore membrane did not invade neighboring channels at any time over 14 days (Figures 6C1–C2). This further confirms the unique character of cancer cell invasiveness and the significant changes in stromal fibroblasts after their conversion to CAFs.

Similar co-cultures of C4-2 cancer cells with BHPrS1 cells were established, where mCherry-labeled BHPrS1 fibroblasts were plated on the membrane in the upper serpentine channel, and GFP-labeled C4-2 cancer cells were plated on the membrane in the lower straight channel (Figure 6D1). Both C4-2 cancer cells and stromal fibroblasts cells were detected in their neighboring channels as early as 3 days into the co-culture. In high-magnification images, invading C4-2 cancer cells appeared as strands or clusters in the stromal fibroblast channel (Figure 6D2), whereas the infiltrated BHPrS1 fibroblasts were spreading in the tumor channel (Figure 6D3). By Day 17 of the co-culture, C4-2 cancer cells had advanced farther with some cells migrating over a distance larger than 1 mm in the fibroblast channel. The original fibroblast monolayer on the membrane surface, proliferating significantly, expanded into multiple layers covering not only their channel entire inner surfaces but also the surfaces of the cancer cell channel. This is consistent with reported observations that stromal fibroblasts undergo a paralleled stromagenesis during tumorigenesis, becoming CAFs and actively participating in and promoting tumor growth and invasion, resembling tissue responses in wound healing (Dvorak, 1986).

At the completion of the experiment, each channel was washed with its culture medium. The loosely adherent C4-2 cancer cell layer were easily washed off the membrane in the tumor channel, leaving a very small number of C4-2 cells attached to their original plating surface. Since the tumor cell layer remained intact out of the channel, we speculate that, as the co-culture progressed, tumor cell-to-membrane adhesion decreased while cell-to-cell adhesion increased, resulting in the detachment of the cell layer from the membrane. Remarkably, C4-2 cancer cells that had invaded into the stromal fibroblast channel remained adherent to the fibroblast cell layer without being washed out (Figure 6D4, blue color arrows), while the adherent BHPrS1 fibroblasts remained attached in the tumor channel after washing (Figure 6D4, magenta color arrow). The culture medium collected from each channel contained both C4-2 cells and stromal fibroblasts, and both types of cells were viable as confirmed by culturing them for 7 additional days in tissue culture dishes.

### 3.5 Effect of tumor-stroma direct physical contact on tumor cell invasion

**FIGURE 7:**
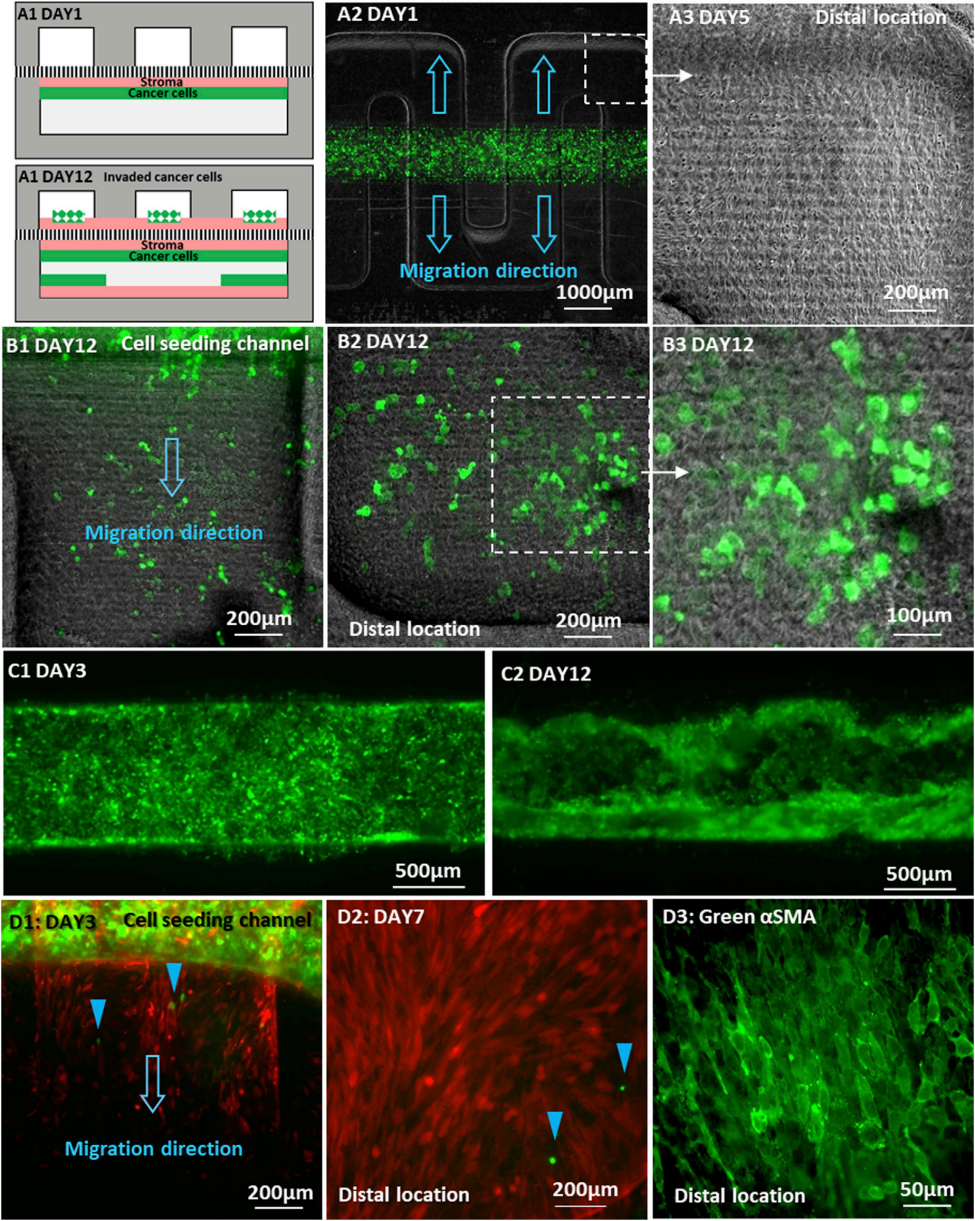
Invasion of cancer cells placed directly on a fibroblast monolayer in the same channel: **(A1)** Schematic locations of C4-2 cancer cells and BHPrS1 fibroblast cells on Day 1 and Day 12 of co-culture; **(A2)** Co-culture of GFP-C4-2 cells and un-labeled BHPrS1 cells with direct physical contact in the lower straight channel while the upper serpentine channel was left empty; **(A3)** BHPrS1 cells in the upper serpentine channel at the distal locations by Day 5; **(B1–B3)** GFP-C4-2 cells at the distal locations of the upper serpentine channel, spreading on top of the BHPrS1 cell layer by Day 12; **(C1)** an initial uniform GFP-C4-2 cell monolayer by Day 3; and **(C2)** GFP-C4-2 cancer cells expanding to the lower surface of the original straight channel by Day 12; **(D1)** BHPrS1 cells first migrated into the empty serpentine channel, followed by 22Rv1 cells indicated by blue color arrows; **(D2)** GFP-22Rv1 cells reached distal location of the serpentine channel by Day 7; **(D3)** BHPrS1 cells stained for green-αSMA.

We further explored the behavior of stromal fibroblasts and tumor cells at the onset of invasion when placed in direct contact. Normal BHPrS1 fibroblasts were plated on the membrane in the lower straight channel to obtain a confluent monolayer. Subsequently, GFP-tagged C4-2 cancer cells were placed directly on top of the fibroblast cell layer establishing 2 cell layers in direct physical contact in the same lower channel. The upper serpentine channel was left empty without cells (Figures 7A1, A2). Culture medium flow was driven through each of the upper and lower channels at a constant flow rate of 30 μL/h, and R1881 treatment was administered only in the lower channel containing both cell types. Stromal fibroblasts were first observed in the upper channel on Day 2 of the co-culture, migrating from the overlapping regions of the straight seeding channel into the upper serpentine channel. In the ensuing few days, the stromal fibroblasts rapidly spread over the entire serpentine channel surface reaching all distal locations (Figure 7A3). Interestingly, in this configuration, the C4-2 cancer cells followed the stromal fibroblasts advancing into the serpentine channel at a very fast pace. Many C4-2 cancer cells were detected at the farthest distal locations in the serpentine channel about 2 mm away from their original straight channel by Day 12. They appeared mostly as single cells or in small cell clusters with some noticeable individual cells, not rounded but spreading on the channel surface (Figures 7B1–B3). The C4-2 cancer cells remaining in their original channel also changed morphologically from a monolayer (Figure 7C1) into a disrupted 3D layer reaching the surface facing the separation membrane of the lower channel that had been covered with stromal fibroblasts (Figure 7C2). This could be attributed to both a high proliferation rate and improved adherence to the channel surface modified by the stromal fibroblasts. In comparison with experiments where stromal fibroblasts and cancer cells were plated on opposite sides of the membrane, the direct physical contact of the cancer cells with fibroblast dramatically increased the number of invading tumor cells along with their migration range and pace.

Similar experiment was repeated with GFP-22Rv1 cancer cells placed directly on top of a confluent monolayer of mCherry-BHPrS1 fibroblasts in the lower straight channel, while the top serpentine channel was left empty without cells. By Day 3, mCherry-fibroblasts were first observed entering the orthogonal channel segments of the serpentine channel, followed by several GFP-22Rv1 cancer cells (Figure 7D1, blue color arrows). In this case, 22Rv1 cells reached the farthest distal location of the serpentine channel by Day 7 (Figure 7D2), exhibiting enhanced invasion and spreading capability in comparison with 22Rv1 co-cultured with BHPrS1 cell on opposite sides of the membrane without direct physical contact (Figure 6B2).

It is worth noting that the stromal fibroblasts were observed to cross the membrane into the empty channel ahead of the cancer cells; no cancer cells were observed at locations beyond regions that had already been covered by fibroblasts. Therefore, it seems that the conversion of fibroblasts to CAFs conditioned them to lead the trailing cancer-cell invasion; a similar behavior was noticed in an experiment *in vitro* of adenoid cystic carcinoma invasion in salivary gland (Li et al., 2016; Attieh et al., 2017). At the completion of the direct-physical contact experiment, fibroblasts in the serpentine channel were stained for αSMA CAF marker. Unlike the experiment with no direct physical contact, the fibroblasts were found to express αSMA throughout the entire channel including at the distal locations (Figure 7D3). This confirms that all migrating fibroblasts in the serpentine channel, regardless of location, were converted into CAFs. It is suspected that the CAF conversion occurred in the original seeding straight channel due to the direct physical contact with tumor cells and, subsequently, the CAFs invaded into the serpentine channel and proliferated. The significantly enhanced effectiveness of the cancer cell invasion strongly suggests that the interaction between the cancer cells and stroma under direct physical contact resulted in simultaneous integration of collaborative communication signals, involving not only secreted factors but additional signaling pathways activated under direct physical contact (Friedl and Alexander, 2011); the latter was impeded by the separation membrane when the cancer cells and stroma were plated on the opposite sides of the membrane.

**FIGURE 8:**
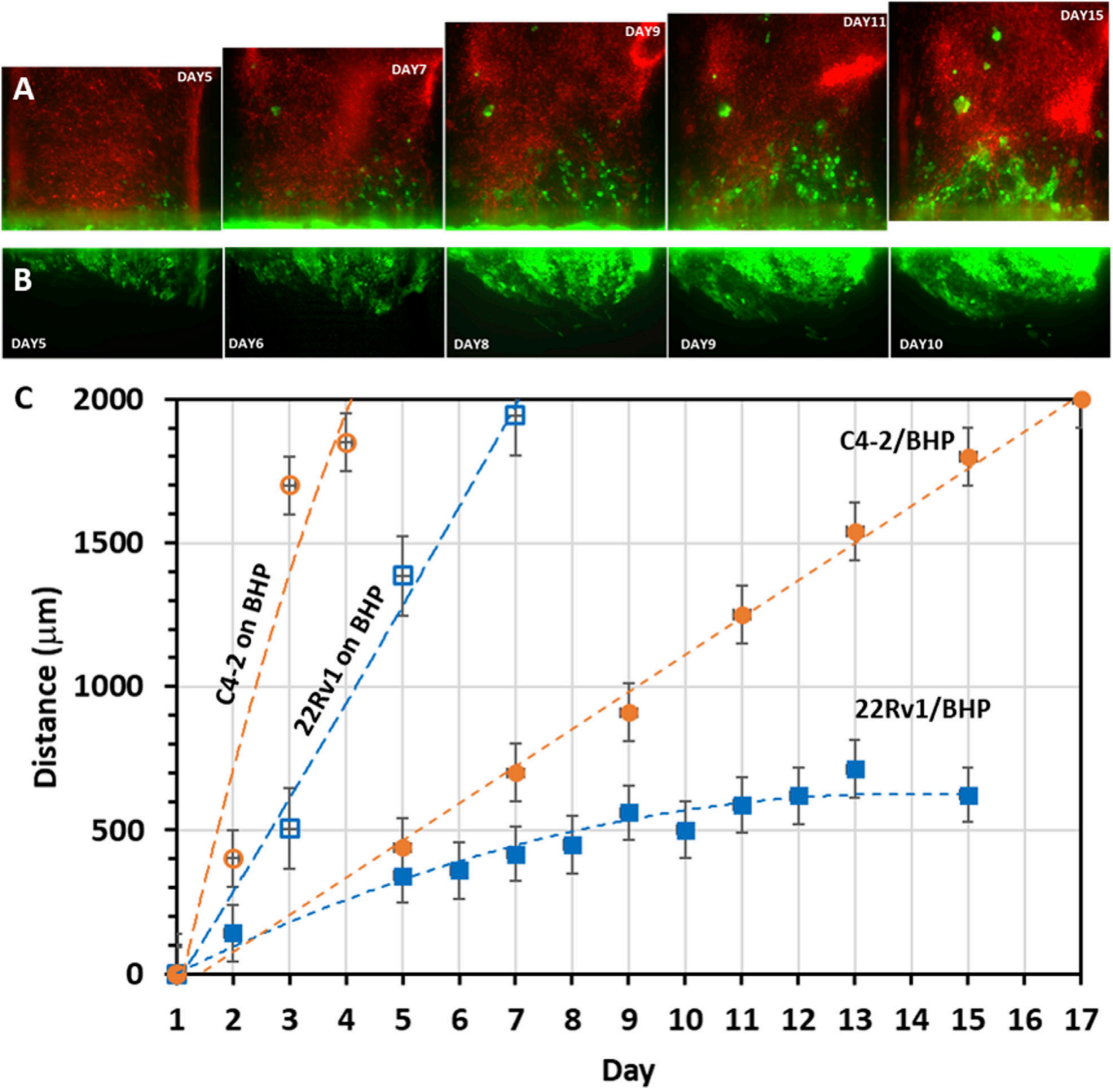
Migration patterns and rates of invading cancer cells: Time-sequence images of cancer cells (seeded in the lower straight channel) invading into neighboring stroma channel (seeded in the upper serpentine channel): **(A)** GFP-C4-2 cells invading into the channel of mCherry-fibroblasts as cell strands and clusters; **(B)** GFP-22Rv1 cells invading the channel of unlabeled fibroblasts as tight cell group. **(C)** Cancer cell migration range based on measured time-dependent distance between the cancer cell front in the fibroblast channel and the edge of the original cancer cell channel. ‘22Rv1/BHP’ or ‘C4-2/BHP’ label indicates co-culture of cancer cells 22Rv1 or C4-2 with stromal BHPrS1 fibroblasts on opposite surfaces of the separation membrane, while ‘22Rv1 on BHP’ or ‘C4-2 on BHP’ label indicates that 22Rv1 or C4-2 cancer cells were placed on top of a stromal BHPrS1 fibroblast monolayer in the same channel. Values in distance were represented as average ± SD of 4 replicates.

We next characterized cancer cell invasion qualitatively and quantitatively by examining its morphological pattern and migration speed, respectively. C4-2 cancer cell invasion started in a strand-like pattern and followed by formation of individual cell clusters with noticeable size increase (Figure 8A). 22Rv1 cancer cell invasion, however, started in a sheet-like pattern with tight cell-cell contacts. As the invasion persisted, the front of the collective cell body did not shift much but rather the cell density within the collective body increased, becoming a sizable 3D dense colony (Figure 8B). The later increase in cluster size is likely a proliferative effect. For experiments where cancer cells were seeded on stromal fibroblast monolayer, scattered individual single cells or cell aggregates, either C4-2 (Figures 7B1, B2) or 22Rv1 (Figure 7D1, D2), appeared to be the pattern. For quantitative analysis, the distance between the most advanced cells, at the invasion front, and edge of the original tumor channel was estimated as a function of time (Figure 8C). Once 22Rv1 cancer cells invaded the neighboring fibroblast layer, by crossing through the membrane pores, they maintained a collective invasion speed of about 0.04 μm/min. In comparison, the invasion speed of C4-2 cancer cells was doubled about 0.08 μm/min. These estimated speeds are within the ranges for cancer collective migrations reported elsewhere (Clark and Vignjevic, 2015; Pandya et al., 2017). A dramatic increase in invasion speed to about 0.2 μm/min or 0.4 μm/min was observed when 22Rv1 or C4-2 cancer cells, respectively, were placed in direct physical contact with stromal fibroblasts prior to the invasion onset. Moreover, the current prostate cancer invasion results are also in good agreement with similar studies in breast cancer asserting that physical contact between cancer cells and fibroblasts promotes CAF conversion and, subsequently, tumor progression (Strell et al., 2019; Sahai et al., 2020).

Tumor cell invasion is accompanied by secretion of cytokines by the tumor cells that modify the tumor microenvironment to drive reorganization of the stroma and, conversely, stromal cells produce cytokines that promote growth and invasion of the tumor. TGFβ, produced by tumor cells is considered as the major driver of CAF conversion in the stroma (Barcellos-de-Souza et al., 2016; Franco et al., 2016). Once the stroma is activated, PDGF produced by the tumor cells act on the PDGF receptors of the stroma cells, causing an increase in PDGFR levels and activity leading to more recruitment and proliferation of CAFs (Primac et al., 2019). Then the PDGF-activated CAFs begin to produce TGFβ as well. TGFβ can in turn act on the tumor cells to drive tumor cell invasion by inducing an epithelial mesenchymal transition (EMT) phenotype [Neri et al., 2017; Nordby et al., 2017]. Another cytokine that is often produced by both tumor cells and stromal cells is TNFα, which stimulates an inflammatory response within the tumor microenvironment by activating resident immune cells (Liubomirski et al., 2019). It has also been implicated in enhancing TGFβ-mediated EMT conversion of tumor cells (Yoshimatsu et al., 2020).

**FIGURE 9:**
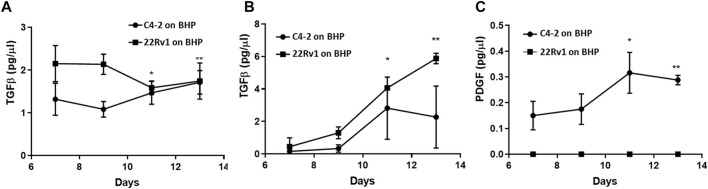
Cytokine production in Prostate Cancer-on-Chip model. Levels of **(A, B)** TGFβ and **(C)** PDGF in conditioned medium measured by ELISA. PDGF was below detectable levels (not shown) in the medium collected from straight channels of both experiments. Conditioned medium was collected every 2 days starting at Day 7, from straight and serpentine channels of 8 µm-pore devices. Straight channel contained BHPrS1 cells and tumor cells, while serpentine channel contained invaded stromal fibroblasts and tumor cells. Error bars = SD; *n* = 4; *<0.05; **<0.001 relative to Day 7 values.

To demonstrate that the Prostate Cancer-on-Chip model recapitulates faithfully the prostate tumor microenvironment, we examined whether any of these cytokines were produced within the Prostate Cancer-on-Chip model using ELISA. Conditioned medium samples collected from experiments with physical contact between tumor and stroma cultures, “C4-2 on BHP” and “22Rv1 on BHP”, were analyzed; samples were collected separately from each channel at 2-day intervals from Day 7 to Day 13 of the co-culture. During this period, both fibroblasts and tumor cells were present in the straight channels, and no significant changes in the number of cells in each population were observed. CAFs had fully occupied the initially empty serpentine channel, while robust tumor cell invasion was occurring as well. The production of TGFβ in the straight channel medium was robustly detected. Higher TGFβ levels were initially produced in “22Rv1 on BHP”, but slightly dropped later, while the levels in “C4-2 on BHP” slightly increased with time (Figure 9A). In the serpentine channel, where most of the cells were CAFs (Figure 7D2), the level of TGFβ in its medium increased with time for the two co-cultures (Figure 9B). This could be attributed to continuous increase in the number of CAFs, over the tested period, producing higher levels of TGFβ. In addition, the number of tumor cells in the serpentine channel also increased over time, from newly invaded tumor cells and cell division of the early invaded tumor cells. Low levels of PDGF were detected only in the medium collected from the serpentine channel of the “C4-2 on BHPrS1” co-culture (Figure 9C), increasing with time presumably due to the observed higher rates in migration and proliferation of the invaded C4-2 cells and CAFs (Figures 7B1–B3). PDGF was below detectable levels in the medium collected from the serpentine channel of the “22Rv1 on BHP” (Figure 9C) and those from the straight channels of both experiments (not shown). TNFα was not detectable in any samples of both experiments (not shown), consistent with the absence of immune cells in this model. The cytokine study exemplified that commonly used bioanalytical techniques and methods can be incorporated in the Prostate Cancer-on-Chip model to yield quantitative data.

## 4 Conclusion

A microfluidics-based Prostate-Cancer-on-Chip (PCoC) has been developed to model the physiology of the human prostate tumor microenvironment. The fabricated microfluidic device consisted of two channels, separated by a porous membrane, for co-culturing of tumor and stromal cells. Guided by numerical simulations, a proper convection-diffusion balance was obtained by fine-tuning the flow field in the microfluidic device allowing not only long-term co-culture maintenance but also continuous communication between the tumor and the surrounding stroma mediated by secreted soluble factors. Tumor cells conversion of normal stromal fibroblasts into CAFs depended on the local concentration of the diffused signaling molecules, which was not uniform featuring strong spatial gradients. CAF conversion was characterized, using known protein markers associated with CAFs, and found to be in good agreement with reported observations. Furthermore, tumor cells invaded the stroma channel and *vice versa* in devices with sufficiently large membrane pore size about 8 µm. Both 22Rv1 and C4-2 cancer cells exhibited collective migration pattern. 22Rv1 cells displayed migration as a tightly packed cluster at 0.04 μm/min whereas C4-2 cells presented migration as cell strands and smaller clusters of cell aggregate at a relatively higher speed of 0.08 μm/min. Remarkably, when cancer cells were placed in direct physical contact with stromal fibroblasts, replicating the native tumor microenvironment, tumor invasion became much more aggressive. Led and promoted by the stromal fibroblasts, trailing cancer cells were found to spread farther and faster at a speed of about 0.2 μm/min and 0.4 μm/min for 22Rv1 and C4-2 cancer cells, respectively. Production of TGFβ and PDGF cytokines known to drive CAF conversion and increase tumor invasion were detected in the conditioned medium collected from the respective channels.

In summary, the newly developed PCoC was found to faithfully replicate the tumor microenvironment of prostate cancer *in vivo* and, thus, providing a clinically relevant *in vitro* human prostate tumor model. It is a promising basic research tool for dissecting interactions between the tumor and its surrounding stroma and elucidating the underlying biological mechanisms. It can also be applied in clinical research for novel-target drug discovery and development overcoming the persistent barriers associated with traditional cell cultures and animal models.

## Data Availability

The original contributions presented in the study are included in the article/Supplementary material, further inquiries can be directed to the corresponding author.

## References

[B1] Almagro J. Messal H. A. Elosegui-Artola A. Rheenen J. V. Behrens A. (2022). Tissue architecture in tumor initiation and progression. Trends Cancer 8, 494–505. 10.1016/j.trecan.2022.02.007 35300951

[B2] Andrews M. G. Kriegstein A. R. (2022). Challenges of organoid research. Annu. Rev. Neurosci. 45, 23–39. 10.1146/annurev-neuro-111020-090812 34985918 PMC10559943

[B3] Angaravati N. Kurniasari C. R. Damayanti K. Cahyanti T. Widodo I. Ghozali A. (2017). Histochemical and immunohistochemical study of α-SMA, collagen, and PCNA in epithelial ovarian neoplasm. Asian Pac. J. Cancer Prev. 18, 667–671. 10.22034/APJCP.2017.18.3.667 28440973 PMC5464482

[B4] Attieh Y. Clark A. G. Grass C. Richon S. Pocard M. Mariani P. (2017). Cancer-associated fibroblasts lead tumor invasion through integrin-β3–dependent fibronectin assembly. J. Cell Biol. 216 (11), 3509–3520. 10.1083/jcb.201702033 28931556 PMC5674886

[B5] Baptista L. S. Porrini C. Kronemberger G. S. Kelly D. J. Perrault C. M. (2022). 3D organ-on-a-chip: the convergence of microphysiological systems and organoids. Front. Cell Dev. Biol. 10, 1043117. 10.3389/fcell.2022.1043117 36478741 PMC9720174

[B6] Barcellos-de-Souza P. Comito G. Pons-Segura C. Taddei M. L. Gori V. Becherucci V. (2016). Mesenchymal stem cells are recruited and activated into carcinoma-associated fibroblasts by prostate cancer microenvironment-derived TGF-β1. Stem Cells 34 (10), 2536–2547. 10.1002/stem.2412 27300750

[B7] Beacham D. A. Cukierman E. S. (2005). Stromagenesis: the changing face of fibroblastic microenvironments during tumor progression. Seminars Cancer Biol. 15, 329–341. 10.1016/j.semcancer.2005.05.003 15970443

[B8] Berger P. L. Frank S. B. Schulz V. V. Nollet E. A. Edick M. J. Holly B. (2014). Transient induction of ING4 by Myc drives prostate epithelial cell differentiation and its disruption drives prostate tumorigenesis. Cancer Res. 74 (12), 3357–3368. 10.1158/0008-5472.can-13-3076 24762396 PMC4066454

[B9] Bharath Rao K. Malathi N. Narashiman S. Rajan S. T. (2014). Evaluation of myofibroblasts by expression of alpha smooth muscle actin: a marker in fibrosis, dysplasia and carcinoma. J. Clin. Diagnostic Res. 8 (4), ZC14–ZC17. 10.7860/JCDR/2014/7820.4231 PMC4064839 24959509

[B10] Castelló-Cros R. Cukierman E. Stromagenesis D. (2009). Stromagenesis during tumorigenesis: characterization of tumor-associated fibroblasts and stroma-derived 3D matrices. Methods Mol. Biol. Clift. N.J.) 522, 275–305. 10.1007/978-1-59745-413-1_19 PMC2670062 19247611

[B11] Cattrini C. Barboro P. Rubagotti A. Zinoli L. Zanardi E. Capaia M. (2020). Integrative analysis of periostin in primary and advanced prostate cancer. Transl. Oncol. 13 (7), 100789. 10.1016/j.tranon.2020.100789 32416542 PMC7248449

[B12] Chung L. W. K. Baseman A. Assikis V. Zhau H. E. (2005). Molecular insights into prostate cancer progression: the missing link of tumor microenvironment. J. Urology, 173. 10.3389/fcell.2023.1089068 15592017

[B13] Chung L. W. K. Hsieh C. L. Law A. Sung S. Y. Gardner T. A. Egawa M. (2003). New targets for therapy in prostate cancer: modulation of stromal-epithelial interactions. Urology 62, 44–54. 10.1016/S0090-4295(03)00796-9 14607217

[B14] Clark A. G. Vignjevic D. M. (2015). Modes of cancer cell invasion and the role of the microenvironment. Curr. Opin. Cell Biol. 36, 13–22. 10.1016/j.ceb.2015.06.004 26183445

[B15] Clark A. K. Taubenberger A. V. Taylor R. A. Niranjan B. Chea Z. Y. Zotenko E. (2013). A bioengineered microenvironment to quantitatively measure the tumorigenic properties of cancer-associated fibroblasts in human prostate cancer. Biomaterials 34, 4777–4785. 10.1016/j.biomaterials.2013.03.005 23562048

[B16] Dorafshan S. Razmi M. Safaei S. Gentilin E. Madjd Z. Ghods R. (2022). Periostin: biology and function in cancer. Cancer Cell Int. 22, 315. 10.1186/s12935-022-02714-8 36224629 PMC9555118

[B17] Duzagac F. Saorin G. Memeo L. Canzonieri V. Rizzolio F. (2021). Microfluidic organoids‐on‐a‐chip: quantum leap in cancer research. Cancers 13, 737. 10.3390/cancers13040737 33578886 PMC7916612

[B18] Dvorak H. D. (1986). Tumors: wounds that do not heal. Similarities between tumor stroma generation and wound healing. N. Engl. J. Med., 315. 3537791 10.1056/NEJM198612253152606

[B19] Ewart L. Apostolou A. Briggs S. A. Carman C. V. Chaff J. T. Heng A. R. (2022). Performance assessment and economic analysis of a human liver-chip for predictive toxicology. Commun. Med. 2, 154. 10.1038/s43856-022-00209-1 36473994 PMC9727064

[B20] Fang M. Yuan J. Peng C. Li Y. (2014). Collagen as a double-edged sword in tumor progression. Tumor Biol. 35, 2871–2882. 10.1007/s13277-013-1511-7 PMC3980040 24338768

[B21] Fong E. L. S. Wan X. Yang J. Morgado M. Mikos A. G. Harrington D. A. (2016). A 3D *in vitro* model of patient-derived prostate cancer xenograft for controlled interrogation of *in vivo* tumor-stromal interactions. Biomaterials 77, 164–172. 10.1016/j.biomaterials.2015.10.059 26599623 PMC4684431

[B22] Francis I. Shrestha J. Paudel K. R. Hansbro P. M. Warkiani M. E. Saha S. C. (2022). Recent advances in lung-on-a-chip models. Drug Discov. Today 27, 2593–2602. 10.1016/j.drudis.2022.06.004 35724916

[B23] Francis K. Palsson B. O. (1997). Effective intercellular communication distances are determined by the relative time constants for cyto/chemokine secretion and diffusion. Proc. Natl. Acad. Sci. U. S. A. 94 (23), 12258–12262. 10.1073/pnas.94.23.12258 9356436 PMC24899

[B24] Franco O. E. Shaw A. K. Strand D. W. Hayward S. W. (2010). Cancer associated fibroblasts in cancer pathogenesis. Seminars Cell Dev. Biol. 21, 33–39. 10.1016/j.semcdb.2009.10.010 PMC2823834 19896548

[B25] Franco O. E. Tyson D. R. Konvinse K. C. Udyavar A. R. Estrada L. Quaranta V. (2016). Altered TGF-α/β signaling drives cooperation between breast cancer cell populations. FASEB J. 30 (10), 3441–3452. 10.1096/fj.201500187RR 27383183 PMC5024699

[B26] Friedl P. Alexander S. (2011). Cancer invasion and the microenvironment: plasticity and reciprocity. Cell 147 (5), 992–1009. 10.1016/j.cell.2011.11.016 22118458

[B27] Gillard M. Javier R. Ji Y. Zheng S. L. Xu J. Brendler C. B. (2018). Elevation of stromal-derived mediators of inflammation promote prostate cancer progression in african-American men. Cancer Res. 78, 6134–6145. 10.1158/0008-5472.CAN-17-3810 30181178

[B28] Glentis A. Oertle P. Mariani P. Chikina A. Marjou F. E. Attieh Y. (2017). Cancer-associated fibroblasts induce metalloprotease-independent cancer cell invasion of the basement membrane. Nat. Commun. 8, 924. 10.1038/s41467-017-00985-8 29030636 PMC5640679

[B29] Goel H. L. Li J. Kogan S. Languino L. R. (2008). Integrins in prostate cancer progression. Endocrine-Related Cancer 15, 657–664. 10.1677/erc-08-0019 18524948 PMC2668544

[B30] González-González L. Alonso J. P. (2018). Periostin: a matricellular protein with multiple functions in cancer development and progression. Front. Oncol. 8, 225. 10.3389/fonc.2018.00225 29946533 PMC6005831

[B31] Hahn A. W. Siddiqui B. A. Leo J. Dondossola E. Basham K. J. Miranti C. K. (2023). Cancer cell–extrinsic roles for the androgen receptor in prostate cancer. Endocrinology 164 (6), bqad078. 10.1210/endocr/bqad078 37192413 PMC10413433

[B32] Hall C. L. Dubyk C. W. Riesenberger T. A. Shein D. Keller E. T. Golen K. L. V. (2008). Type I collagen receptor (α2β1) signaling promotes prostate cancer invasion through RhoC GTPase. Neoplasia 10, 797–803. 10.1593/neo.08380 18670640 PMC2481569

[B33] Hayward S. W. Dahiya R. Cunha G. R. Bartek J. Deshpande N. Narayan P. (1995). Establishment and characterization of an immortalized but non-transformed human prostate epithelial cell line: BPH-1. Vitro Cell. Dev. Biol. - Animal 31, 14–24. 10.1007/BF02631333 7535634

[B34] Hayward S. W. Haughney P. C. Rosen M. A. Greulich K. M. Weier H. U. Dahiya R. (1998). Interactions between adult human prostatic epithelium and rat urogenital sinus mesenchyme in a tissue recombination model. Differentiation 63, 131–140. 10.1046/j.1432-0436.1998.6330131.x 9697307

[B35] Hemelryk A. V. Weerden W. M. V. (2020). Novel patient-derived 3D culture models to guide clinical decision-making in prostate cancer. Curr. Opin. Endocr. Metabolic Res., 10.

[B36] Henshall S. M. Quinn D. I. Lee C. S. Head D. R. Golovsky D. Brenner P. C. (2001). Altered expression of androgen receptor in the malignant epithelium and adjacent stroma is associated with early relapse in prostate cancer. Cancer Res., 61. 11212224

[B37] Ingber D. E. (2022). Human organs-on-chips for disease modelling, drug development and personalized medicine. Nat. Rev. Genet. 23, 467–491. 10.1038/s41576-022-00466-9 35338360 PMC8951665

[B38] Jeong S. Y. Lee J. H. Shin Y. Chung S. Kuh H. J. (2016). Co-culture of tumor spheroids and fibroblasts in a collagen matrix-incorporated microfluidic chip mimics reciprocal activation in solid tumor microenvironment. PLoS ONE 11, 0159013. 10.1371/journal.pone.0159013 PMC4938568 27391808

[B39] Jiang L. Ivich F. Tahsin S. Tran M. Frank S. B. Miranti C. K. (2019). Human stroma and epithelium Co-culture in a microfluidic model of a human prostate gland. Biomicrofluidics 13, 064116. 10.1063/1.5126714 31768202 PMC6867939

[B40] Kahane H. Sharp J. W. Shuman G. B. Dasilva G. Epstein J. I. (1995). Utilization of high molecular weight cytokeratin on prostate needle biopsies in an independent laboratory. Urology 45, 981–986. 10.1016/S0090-4295(99)80118-6 7539563

[B41] Kii I. Nishiyama T. Kudo A. (2016). Periostin promotes secretion of fibronectin from the endoplasmic reticulum. Biochem. Biophysical Res. Commun. 470, 888–893. 10.1016/j.bbrc.2016.01.139 26820539

[B42] Kim C. K. Lee Y. R. Ong L. Gold M. Kalali A. Sarkar (2022). Alzheimer’s disease: key insights from two decades of clinical trial failures. J. Alzheimer’s Dis. 87 (1), 83–100. 10.3233/JAD-215699 35342092 PMC9198803

[B43] Kim J. Koo B. K. Knoblich J. A. (2020). Human organoids: model systems for human biology and medicine. Nat. Rev. Mol. Cell Biol. 21, 571–584. 10.1038/s41580-020-0259-3 32636524 PMC7339799

[B44] Kim J. J. Yin B. Christudass C. S. Terada N. Rajagopalan K. Fabry B. (2013). Acquisition of paclitaxel resistance is associated with a more aggressive and invasive phenotype in prostate cancer. J. Cell. Biochem. 114, 1286–1293. 10.1002/jcb.24464 23192682 PMC4211414

[B45] King T. E. Pawar S. C. Majuta L. Sroka I. C. Wynn D. Demetriou M. C. (2008). The role of alpha 6 integrin in prostate cancer migration and bone pain in a novel xenograft model. PLoS ONE 3, e3535. 10.1371/journal.pone.0003535 18958175 PMC2570216

[B46] Ko C. J. Huang C. C. Lin H. Y. Juan C. P. Lan S. W. Shyu H. Y. (2015). Androgen-induced TMPRSS2 activates matriptase and promotes extracellular matrix degradation, prostate cancer cell invasion, tumor growth, and metastasis. Cancer Res. 75, 2949–2960. 10.1158/0008-5472.CAN-14-3297 26018085

[B47] Kunju L. P. Mehra R. Snyder M. Shah R. B. (2006). Prostate-specific antigen, high-molecular-weight cytokeratin (clone 34βE12), and/or p63: an optimal immunohistochemical panel to distinguish poorly differentiated prostate adenocarcinoma from urothelial carcinoma. Am. J. Clin. Pathology 125, 675–681. 10.1309/v1ry91nkx5arw2q5 16707367

[B48] Labernadie A. Kato T. Brugués A. Serra-Picamal X. Derzsi S. Arwert E. (2017). A mechanically active heterotypic E-cadherin/N-cadherin adhesion enables fibroblasts to drive cancer cell invasion. Nat. Cell Biol. 19 (3), 224–237. 10.1038/ncb3478 28218910 PMC5831988

[B49] Lang S. H. Hyde C. Reid I. N. Hitchcock I. S. Hart C. A. Bryden A. A. G. (2002). Enhanced expression of vimentin in motile prostate cell lines and in poorly differentiated and metastatic prostate carcinoma. Prostate 52, 253–263. 10.1002/pros.10088 12210485

[B50] Li J. Jia Z. Kong J. Zhang F. Fang S. Li X. (2016). Carcinoma-associated fibroblasts lead the invasion of salivary gland adenoid cystic carcinoma cells by creating an invasive Track. PLoS ONE 11, 0150247. 10.1371/journal.pone.0150247 PMC4782997 26954362

[B51] Liu X. Fang J. Huang S. Wu X. Xie X. Wang J. (2021). Tumor-on-a-Chip: from bioinspired design to biomedical application. Microsystems Nanoeng. 7, 50. 10.1038/s41378-021-00277-8 PMC8433302 34567763

[B52] Liubomirski Y. Lerrer S. Meshel T. Rubinstein-Achiasaf L. Morein D. Wiemann S. (2019). Tumor-stroma-inflammation networks promote pro-metastatic chemokines and aggressiveness characteristics in triple-negative breast cancer. Front. Immunol. 10 (APR), 757. 10.3389/fimmu.2019.00757 31031757 PMC6473166

[B53] Lopaczynski W. Hruszkewycz A. M. Lieberman R. P. (2001). Preprostatectomy: a clinical model to study stromal-epithelial interactions. Urology 57, 194–199. 10.1016/s0090-4295(00)00973-0 11295626

[B54] Lu J. G. Lo E. T. Williams C. Ma B. Sherrod A. E. Xiao G. Q. (2023). Expression of high molecular weight cytokeratin—a novel feature of aggressive and innate hormone-refractory prostatic adenocarcinoma. Prostate 83, 462–469. 10.1002/pros.24478 36576021

[B55] Lucas J. M. Heinlein C. Kim T. Hernandez S. A. Malik M. S. True L. D. (2014). The androgen-regulated protease TMPRSS2 activates a proteolytic cascade involving components of the tumor microenvironment and promotes prostate cancer metastasis. Cancer Discov. 4, 1310–1325. 10.1158/2159-8290.CD-13-1010 25122198 PMC4409786

[B56] Multhaupt H. A. B. Fessler J. N. Warhol M. J. (2000). Loss of high-molecular-weight cytokeratin antigenicity in prostate tissue obtained by transurethral resections. Archives Pathology Laboratory Med. 124, 1764–1767. 10.5858/2000-124-1764-lohmwc 11100054

[B57] Nakazawa M. Paller C. Kyprianou N. (2017). Mechanisms of therapeutic resistance in prostate cancer. Curr. Oncol. Rep. 19, 13. 10.1007/s11912-017-0568-7 28229393 PMC5812366

[B58] Neri S. Miyashita T. Hashimoto H. Suda Y. Ishibashi M. Kii H. (2017). Fibroblast-led cancer cell invasion is activated by epithelial–mesenchymal transition through platelet-derived growth factor BB secretion of lung adenocarcinoma. Cancer Lett. 395, 20–30. 10.1016/j.canlet.2017.02.026 28286261

[B59] Nollet E. A. Cardo-Vila M. Ganguly S. S. Tran J. D. Schulz V. v. Cress A. (2020). Androgen receptor-induced integrin α6β1 and Bnip3 promote survival and resistance to PI3K inhibitors in castration-resistant prostate cancer. Oncogene 39 (31), 5390–5404. 10.1038/s41388-020-1370-9 32565538 PMC7395876

[B60] Nurmik M. Ullmann P. Rodriguez F. Haan S. Letellier E. (2020). Search of definitions: cancer-associated fibroblasts and their markers. Int. J. Cancer, 146. 10.1002/ijc.32193 PMC6972582 30734283

[B61] Nuzzo P. V. Rubagotti A. Zinoli L. Ricci F. Salvi S. Boccardo S. (2012). Prognostic value of stromal and epithelial periostin expression in human prostate cancer: correlation with clinical pathological features and the risk of biochemical relapse or death. BMC Cancer 12, 625. 10.1186/1471-2407-12-625 23273263 PMC3553030

[B62] Olumi A. F. Grossfeld G. D. Hayward S. W. Carroll P. R. Tlsty T. D. Cunha G. R. (1999). Carcinoma-associated fibroblasts direct tumor progression of initiated human prostatic epithelium. Cancer Res. 59, 5002–5011. 10.1186/bcr138 10519415 PMC3300837

[B63] Orimo A. Weinberg R. A. (2007). Heterogeneity of stromal fibroblasts in tumor. Cancer Biol. Ther. 6, 618–619. 10.4161/cbt.6.4.4255 18027438

[B64] Pandya P. Orgaz J. L. Sanz-Moreno V. (2017). Modes of invasion during tumour dissemination. Mol. Oncol. 11, 5–27. 10.1002/1878-0261.12019 28085224 PMC5423224

[B65] Parikh J. G. Kulkarni A. Johns C. (2014). α-Smooth muscle actin-positive fibroblasts correlate with poor survival in hepatocellular carcinoma. Oncol. Lett. 7, 573–575. 10.3892/ol.2013.1720 24396490 PMC3881692

[B66] Parker D. C. Folpe A. L. Bell J. Oliva E. Young R. H. Cohen C. (2003). Potential utility of uroplakin III, thrombomodulin, high molecular weight cytokeratin, and cytokeratin 20 in noninvasive, invasive, and metastatic urothelial (transitional cell) carcinomas. Am. J. Surg. Pathology 27, 1–10. 10.1097/00000478-200301000-00001 12502922

[B67] Pederzoli F. Raffo M. Pakula H. Ravera F. Nuzzo P. V. Loda M. (2023). Stromal cells in prostate cancer pathobiology: friends or foes? Br. J. Cancer, 128. 10.1038/s41416-022-02085-x PMC10006214 36482187

[B68] Pickup M. W. Mouw J. K. Weaver V. M. (2014). The extracellular matrix modulates the hallmarks of cancer. EMBO Rep. 15, 1243–1253. 10.15252/embr.201439246 25381661 PMC4264927

[B69] Primac I. Maquoi E. Blacher S. Heljasvaara R. van Deun J. Smeland H. Y. H. (2019). Stromal integrin α11 regulates PDGFRβ signaling and promotes breast cancer progression. J. Clin. Investigation 129 (11), 4609–4628. 10.1172/JCI125890 PMC6819106 31287804

[B70] Qin X. Yan M. Zhang J. Wang X. Shen Z. Lv Z. (2016). TGFβ3-mediated induction of Periostin facilitates head and neck cancer growth and is associated with metastasis. Sci. Rep. 6, 20587. 10.1038/srep20587 26857387 PMC4746667

[B71] Rabinovitz I. Nagle R. B. Cress A. E. (1995). Integrin Α6 expression in human prostate carcinoma cells is associated with a migratory and invasive phenotype *in vitro* and *in vivo* . Clin. Exp. Metastasis 13, 481–491. 10.1007/BF00118187 7586806 PMC2846819

[B72] Rao K. B. Malathi N. Narashiman S. Rajan S. T. (2014). Evaluation of myofibroblasts by expression of alpha smooth muscle actin: a marker in fibrosis, dysplasia and carcinoma. J. Clin. Diagnostic Res. 8, ZC14–ZC17. 10.7860/JCDR/2014/7820.4231 PMC4064839 24959509

[B73] Raskov H. Orhan A. Gaggar S. Gögenur I. (2021). Cancer-associated fibroblasts and tumor-associated macrophages in cancer and cancer immunotherapy. Front. Oncol. 11, 668731. 10.3389/fonc.2021.668731 34094963 PMC8172975

[B74] Rhee H. W. Zhau H. E. Pathak S. Multani A. S. Pennanen S. Visakorpi T. (2001). Permanent phenotypic and genotypic changes of prostate cancer cells cultured in a three-dimensional rotating-wall vessel. Vitro Cell. Dev. Biol. - Animal 37, 127. 10.1290/1071-2690(2001)037<0127:PPAGCO>2.0.CO;2 11370803

[B75] Rubenstein C. S. Gard J. M. C. Wang M. McGrath J. E. Ingabire N. Hinton J. P. (2019). Gene editing of α6 integrin inhibits muscle invasive networks and increases cell–cell biophysical properties in prostate cancer. Cancer Res. 79, 4703–4714. 10.1158/0008-5472.CAN-19-0868 31337652 PMC6750953

[B76] Sahai E. Astsaturov I. Cukierman E. DeNardo D. G. Egeblad M. Evans R. M. (2020). A framework for advancing our understanding of cancer-associated fibroblasts. Nat. Rev. Cancer, 20. 10.1038/s41568-019-0238-1 PMC7046529 31980749

[B77] Sasaki T. Franco O. E. Hayward S. W. (2017). Interaction of prostate carcinoma-associated fibroblasts with human epithelial cell lines *in vivo* . Differentiation 96, 40–48. 10.1016/j.diff.2017.07.002 28779656 PMC5669818

[B101] Savinell J. M. Lee G. M. Palsson B. O. (1989). On the orders of magnitude of epigenic dynamics and monoclonal antibody production. Bioprocess Eng. 4 (5). 10.1007/BF00369177

[B78] Seyhan A. A. (2019). Lost in translation: the valley of death across preclinical and clinical divide – identification of problems and overcoming obstacles. Transl. Med. Commun. 4, 18. 10.1186/s41231-019-0050-7

[B79] Stensland K. D. Daignault-Newton S. Skolarus T. A. (2022). Considerations in the analysis of clinical trial failure. J. Urology, 207. 10.1097/JU.0000000000002206 34448630

[B80] Stensland K. D. DePorto K. Ryan J. Kaffenberger S. Reinstatler L. S. Galsky M. (2021). Estimating the rate and reasons of clinical trial failure in urologic oncology. Urologic Oncol. Seminars Orig. Investigations 39, 154–160. 10.1016/j.urolonc.2020.10.070 PMC7902467 33257221

[B81] Storch K. N. Taatjes D. J. Bouffard N. A. Locknar S. Bishop N. M. Langevin H. M. (2007). Alpha smooth muscle actin distribution in cytoplasm and nuclear invaginations of connective tissue fibroblasts. Histochem. Cell Biol. 127, 523–530. 10.1007/s00418-007-0275-9 17310383

[B82] Strell C. Paulsson J. Jin S. B. Tobin N. P. Mezheyeuski A. Roswall P. (2019). Impact of epithelial–stromal interactions on peritumoral fibroblasts in ductal carcinoma *in situ* . J. Natl. Cancer Inst. 111, 983–995. 10.1093/JNCI/DJY234 30816935 PMC6748730

[B83] Thompson T. C. Southgate J. Kitchener G. Land H. (1989). Multistage carcinogenesis induced by ras and Myc oncogenes in a reconstituted organ. Cell 56, 917–930. 10.1016/0092-8674(89)90625-9 2538247

[B84] Tian Y. Choi C. H. Li Q. K. Rahmatpanah F. B. Chen X. Kim S. R. (2015). Overexpression of periostin in stroma positively associated with aggressive prostate cancer. PLoS ONE 10, 0121502. 10.1371/journal.pone.0121502 PMC4362940 25781169

[B85] Tischler V. Fritzsche F. R. Wild P. J. Stephan C. Seifert H. H. Riener M. O. (2010). Periostin is up-regulated in high grade and high stage prostate cancer. BMC Cancer 10, 273. 10.1186/1471-2407-10-273 20534149 PMC2903527

[B86] Toivanen R. Shen M. M. (2017). Prostate organogenesis: tissue induction, hormonal regulation and cell type specification. Cambridge: Development, 144. 10.1242/dev.148270 PMC5399670 28400434

[B87] Tsunoda T. Furusato B. Takashima Y. Ravulapalli S. Dobi A. Srivastava S. (2009). The increased expression of periostin during early stages of prostate cancer and advanced stages of cancer stroma. Prostate 69, 1398–1403. 10.1002/pros.20988 19479898

[B88] Tuxhorn J. A. Ayala G. E. Smith M. J. Smith V. C. Dang T. D. Rowley D. R. (2002). Reactive stroma in human prostate cancer: induction of myofibroblast phenotype and extracellular matrix remodeling. Clin. Cancer Res. 8, 2912–2923. 12231536

[B89] Vermeulen L. de Sousa E Melo F. van der Heijden M. Cameron K. de Jong J. H. Borovski T. (2010). Wnt activity defines colon cancer stem cells and is regulated by the microenvironment. Nat. Cell Biol. 12 (5), 468–476. 10.1038/ncb2048 20418870

[B90] Vunjak-Novakovic G. Ronaldson-Bouchard K. Radisic M. (2021). Organs-on-a-Chip models for biological research. Cell, 184. 10.1016/j.cell.2021.08.005 PMC8417425 34478657

[B91] Wang R. Lewis M. S. Lyu J. Zhau H. E. Pandol S. J. Chung L. W. K. (2020). Cancer-stromal cell fusion as revealed by fluorescence protein tracking. Prostate 80, 274–283. 10.1002/pros.23941 31846114 PMC6949378

[B92] Wang R. Sun X. Wang C. Y. Hu P. Chu C. Y. Liu S. (2012). Spontaneous cancer-stromal cell fusion as a mechanism of prostate cancer androgen-independent progression. PLoS ONE 7, e42653. 10.1371/journal.pone.0042653 22880071 PMC3411834

[B93] Wang R. Xu J. Juliette L. Castilleja A. Love J. Sung S. Y. (2005). Three-dimensional Co-culture models to study prostate cancer growth, progression, and metastasis to bone. Seminars Cancer Biol. 15, 353–364. 10.1016/j.semcancer.2005.05.005 15982899

[B94] Wikström P. Marusic J. Stattin P. Bergh A. (2009). Low stroma androgen receptor level in normal and tumor prostate tissue is related to poor outcome in prostate cancer patients. Prostate 69, 799–809. 10.1002/pros.20927 19189305

[B95] Yang X. J. Lecksell K. Gaudin P. Epstein J. I. (1999). Rare expression of high-molecular-weight cytokeratin in adenocarcinoma of the prostate gland. A study of 100 cases of metastatic and locally advanced prostate cancer. Am. J. Surg. Pathology 23, 147–152. 10.1097/00000478-199902000-00002 9989840

[B96] Yoshimatsu Y. Wakabayashi I. Kimuro S. Takahashi N. Takahashi K. Kobayashi M. (2020). TNF‐α enhances TGF‐β‐induced endothelial‐to‐mesenchymal transition via TGF‐β signal augmentation. Cancer Sci. 111 (7), 2385–2399. 10.1111/cas.14455 32385953 PMC7385392

[B97] Yoshioka N. Fuji S. Shimakage M. Kodama K. Hakura A. Yutsudo M. (2002). Suppression of anchorage-independent growth of human cancer cell lines by the TRIF52/periostin/OSF-2 gene. Exp. Cell Res. 279, 91–99. 10.1006/excr.2002.5590 12213217

[B98] Young M. E. Carroad P. A. Bell R. L. (1980). Estimation of diffusion coefficients of proteins. Biotechnol. Bioeng. 22 (5), 947–955. 10.1002/bit.260220504

[B99] Yuki K. Cheng N. Nakano M. Kuo C. J. (2020). Organoid models of tumor immunology. Trends Immunol., 41. 10.1016/j.it.2020.06.010 PMC7416500 32654925

[B100] Zhao Y. Yan Q. Long X. Chen X. Wang Y. (2008). Vimentin affects the mobility and invasiveness of prostate cancer cells. Cell Biochem. Funct. 26, 571–577. 10.1002/cbf.1478 18464297

