# Corrigendum: Microfluidic-based human prostate-cancer-on-chip

**DOI:** 10.3389/fbioe.2024.1520130

**Published:** 2024-11-12

**Authors:** Linan Jiang, Hunain Khawaja, Shekha Tahsin, Tanjia A. Clarkson, Cindy K. Miranti, Yitshak Zohar

**Affiliations:** ^1^ Department of Aerospace and Mechanical Engineering, Tucson, AZ, United States; ^2^ Cancer Biology Graduate Interdisciplinary Program, Tucson, AZ, United States; ^3^ College of Sciences, Tucson, AZ, United States; ^4^ Department of Molecular and Cellular Biology, Tucson, AZ, United States; ^5^ University of Arizona Cancer Center, University of Arizona, Tucson, AZ, United States

**Keywords:** organ-on-chip, microfluidics, prostate cancer, stromal fibroblasts, cancer-associated fibroblasts (CAFs), prostate tumor invasion, tumor microenvironment

In the published article, there was an error in the **Funding** statement. The Funding statement was incorrectly written as: “The author(s) declare financial support was received for the research, authorship, and/or publication of this article. LJ, HK, ST, TC, CM, and YZ were supported by funding from NIH/NCI MPI R01 CA254200 as part of the Tissue Engineering Consortium and TACMASR by P30 CA023075 UACC support grant. TC acknowledges partial support by the NIH/NCI U54CA143924 for the Partnership of Native American Cancer Prevention (NACP).” The correct **Funding** statement appears below:

